# Pregnancy and perinatal outcomes after modified natural cycle-frozen embryo transfers according to size of the dominant follicle on the hCG trigger day

**DOI:** 10.1093/hropen/hoaf047

**Published:** 2025-07-16

**Authors:** Jie Zhang, Shuwen Qiu, Hongyuan Gao, Xiaoyan Mao, Yi Guo, Ling Wu

**Affiliations:** Department of Assisted Reproduction, Shanghai Ninth People’s Hospital, Shanghai Jiao Tong University School of Medicine, Shanghai, China; Department of Assisted Reproduction, Shanghai Ninth People’s Hospital, Shanghai Jiao Tong University School of Medicine, Shanghai, China; Department of Assisted Reproduction, Shanghai Ninth People’s Hospital, Shanghai Jiao Tong University School of Medicine, Shanghai, China; Department of Assisted Reproduction, Shanghai Ninth People’s Hospital, Shanghai Jiao Tong University School of Medicine, Shanghai, China; Center for Reproductive Medicine, Shanghai First Maternity and Infant Hospital, School of Medicine, Tongji University, Shanghai, China; Department of Assisted Reproduction, Shanghai Ninth People’s Hospital, Shanghai Jiao Tong University School of Medicine, Shanghai, China

**Keywords:** follicle size, modified natural cycle-frozen thawed embryo transfer, live birth rate, pregnancy outcomes, perinatal outcomes

## Abstract

**STUDY QUESTION:**

Is there an association between the dominant follicle size on the day of the hCG ovulation trigger and reproductive, obstetric, and perinatal outcomes in modified natural cycle-frozen embryo transfer (NC-FET) cycles in which intensive luteal phase support (LPS) was utilized?

**SUMMARY ANSWER:**

The dominant follicle size on the day of triggering, ranging from above 10 to 18 mm or larger, was not associated with negative live birth or perinatal outcomes in modified NC-FETs when an intensive LPS was provided.

**WHAT IS KNOWN ALREADY:**

There is growing evidence concerning the optimal timing for triggering final oocyte maturation during IVF ovarian stimulation cycles to obtain mature oocytes. However, a consensus on the ideal follicle size for administering hCG in modified NC-FETs has yet to be established. Interestingly, it has been suggested that the presence of a mature oocyte may not be necessary for FET.

**STUDY DESIGN, SIZE, DURATION:**

A retrospective cohort study was conducted at a university-affiliated reproductive medicine center. The study included women with regular menstrual cycles who underwent autologous modified NC-FETs between 2013 and 2023 for potential analysis.

**PARTICIPANTS/MATERIALS, SETTING, METHODS:**

Patients were categorized into eight groups based on the size of the dominant follicle at the time of hCG administration: <12, 12–12.9, 13–13.9, 14–14.9, 15–15.9, 16–16.9, 17–17.9, and ≥18 mm. The primary outcome measured was the live birth rate (LBR), while secondary outcomes included the rates of positive pregnancy tests, implantation, clinical pregnancy, and pregnancy loss, as well as perinatal and obstetric complications. A generalized estimating equation logistic regression model was employed to account for the clustered nature of the data and to adjust for potential confounding factors. The group with a follicle size ≥18 mm was designated as the reference group in the logistic regression analyses. An intensive LPS, consisting of 40 mg dydrogesterone plus 400 mg vaginal progesterone, was adopted.

**MAIN RESULTS AND THE ROLE OF CHANCE:**

A total of 14 431 cycles that met the inclusion criteria were analyzed. The LBRs were similar across the eight groups. Furthermore, there were no significant differences in LBRs when the reference group was compared to the other follicle-size categories in the unadjusted models. Even after adjusting for several key confounders, the LBRs remained comparable between the study cohorts and the reference controls. Additionally, other reproductive parameters, such as rates of positive pregnancy tests, implantation, clinical pregnancy, and pregnancy loss, showed similar results between the control group and all other groups in both the unadjusted and confounder-adjusted analyses. Finally, pregnancies derived from the other follicle-size groups did not show increased risks of adverse perinatal or obstetric outcomes when compared to the reference group, both before and after adjustment for covariates.

**LIMITATIONS, REASONS FOR CAUTION:**

The primary limitation of the current study lies in its retrospective and single-center design. Future prospective research is needed to confirm our findings. While this represents the largest investigation to date into cycle outcomes related to follicle size at ovulation trigger in modified NC-FETs, the sample sizes in certain subgroups were relatively small, which may limit the statistical power to identify differences among some groups. Therefore, caution is advised in the interpretation of these findings. Additionally, an intensive LPS was used, potentially reducing the external validity of our findings.

**WIDER IMPLICATIONS OF THE FINDINGS:**

The current data suggest that with the administration of an intensive LPS, the hCG trigger can be given across a wide range of follicle sizes, from above 10 to 18 mm or larger in diameter, in modified NC-FETs. This approach not only simplifies patient monitoring but also offers greater flexibility and autonomy in scheduling the date of embryo transfer.

**STUDY FUNDING/COMPETING INTEREST(S):**

This study was supported by the National Natural Science Foundation of China (grant no. 82171685). The authors declare that they have no competing interests.

**TRIAL REGISTRATION NUMBER:**

N/A.

WHAT DOES THIS MEAN FOR PATIENTS? Frozen embryo transfers (FETs) have become indispensable in the field of assisted reproduction. Instead of using artificial hormonally stimulated cycles for endometrial preparation before FET, modified natural cycles use no pharmacological intervention except human chorionic gonadotrophin (hCG) to induce ovulation. However, regular ultrasound monitoring of the follicle size and endometrial thickness is needed to ensure the optimal timing of hCG administration, and there is currently no consensus regarding the optimal timing of this trigger. Due to the limited research in this area, reproductive physicians generally administer hCG when the leading follicle attains a mature size of at least 18 mm. However, a mature oocyte may not be essential for frozen embryo transfers. Moreover, awaiting the follicle to reach a mature size increases the number of clinic visits, and laboratories must be prepared for embryo transfers on any day of the week.

The current large cohort study indicates that when providing an intensive luteal phase support (exogenous progesterone supplementation during the luteal phase), the hCG trigger can be given across a wide range of follicle sizes, from above 10 to 18 mm or larger in diameter, for modified natural cycle-frozen embryo transfers. Ovulation triggering at smaller follicle sizes was not associated with negative reproductive and perinatal outcomes. This not only simplifies patient monitoring but also offers greater flexibility and autonomy in scheduling the date of embryo transfer.

## Introduction

The number of frozen embryo transfers (FETs) has sharply increased over recent decades, and FET has become an indispensable tool in the field of assisted reproduction (Smeenk *et al.*, 2023; Centers for Disease Control and Prevention, 2022). Nonetheless, the optimal endometrial preparation protocol for FET is still under debate (Montagut *et al.*, 2016; Groenewoud *et al.*, 2018). A recent Cochrane review failed to demonstrate the superiority of one cycle regimen for FET over the others in terms of reproductive outcomes (Ghobara *et al.*, 2025).

There are two primary regimens for endometrial preparation in FET: natural cycle-FET (NC-FET) and artificial cycle-FET (AC-FET). AC-FET is the more commonly utilized approach, as it is suitable for both anovulatory and ovulatory women. Furthermore, this protocol provides convenience for both patients and clinicians, as it requires minimal monitoring and offers flexibility in scheduling embryo transfers (Mackens *et al.*, 2017). Nevertheless, the medications used in AC-FET can be costly, and long-term estrogen supplementation carries inherent risks, such as thromboembolic events (Dalsgaard *et al.*, 2022). Most notably, recent evidence has indicated that AC-FET is associated with an increased risk of adverse obstetric complications, including hypertensive disorders of pregnancy, preeclampsia, and postpartum hemorrhage (Ginström Ernstad *et al.*, 2019; von Versen-Höynck *et al.*, 2019; Busnelli *et al.*, 2022). These safety concerns underscore the preference for NC-FET over AC-FET in ovulatory women (Lawrenz *et al.*, 2020; von Versen-Höynck and Griesinger, 2022).

Relative to AC-FET, NC-FET requires intensified clinical visits to determine the time of ovulation. In true NC-FETs, no medication is administered before ovulation, and a spontaneous LH surge is detected by numerous transvaginal ultrasounds and hormone assays. Nonetheless, it remains challenging to accurately detect ovulation owing to the varying definitions of the LH surge (Erden *et al.*, 2022; Lawrenz *et al.*, 2023). Furthermore, awaiting the LH surge results in uncertain FET planning, which may be troublesome for patients and IVF laboratories (Groenewoud *et al.*, 2016). A modified NC-FET, where the ovulation is triggered by hCG, was therefore developed to overcome the difficulties in the identification of the LH surge (Mackens *et al.*, 2020; Mendes Godinho *et al.*, 2024). This approach makes cycles more predictable, but regular ultrasound monitoring of the follicle size and endometrial thickness is needed to ensure the optimal timing of hCG. Despite these measures, early unexpected ovulation still frequently occurs, resulting in cycle cancellations (Groenewoud *et al.*, 2016, 2017).

Currently, there are no standardized criteria for determining the appropriate time of hCG trigger in modified NC-FETs. Typically, clinicians prefer to administer hCG when the leading follicle reaches an average diameter of 17–18 mm, similar to protocols used in IVF ovarian stimulation cycles to obtain mature oocytes (Groenewoud *et al.*, 2013; Alonso-Mayo *et al.*, 2024). However, it is important to note that a mature oocyte may not be necessary for FET (Alonso-Mayo *et al.*, 2024). This raises the question of whether hCG triggering at smaller follicle sizes in modified NC-FETs is associated with adverse cycle outcomes. In light of this context, the present study aimed to investigate the relationship between different follicle sizes on the hCG trigger day with the reproductive, perinatal, and obstetric outcomes of women undergoing modified NC-FETs.

## Materials and methods

### Study design and population

A retrospective cohort study was carried out at the university-affiliated reproductive medicine center, the Department of Assisted Reproduction, Shanghai Ninth People's Hospital, Shanghai Jiao Tong University School of Medicine, Shanghai, China. The study included women with regular menstrual cycles (25–35 days) who underwent autologous modified NC-FET from January 2013 to June 2023. Inclusion criteria were those with only one dominant follicle (>10 mm) visible on the day of the hCG trigger (Lawrenz *et al.*, 2024). Exclusion criteria were as follows: (i) cycles involving exogenous ovarian stimulation, such as hMG or letrozole; (ii) ovulation before hCG administration; (iii) no embryo survival after thawing; (iv) missing data, including hormonal levels on the trigger day, endometrial thickness, or unrecorded follicle size; and (v) information lost to follow-up. Since preimplantation genetic testing for aneuploidy was not available in our center, none of the patients received this technology. Additionally, a freeze-all strategy utilizing vitrification has been routinely implemented in our center (Yang *et al.*, 2019; Zhang *et al.*, 2019a), as FET has been shown to produce comparable pregnancy outcomes to fresh cycles while minimizing the risk of ovarian hyperstimulation syndrome (Chen *et al.*, 2016; Zaat *et al.*, 2021). The study protocol was approved by the hospital ethics committee, and a waiver for informed consent was approved because this was a retrospective study and patient data were anonymous. Participants were categorized into eight groups based on the size of the leading follicle measured on the hCG trigger day: <12, 12–12.9, 13–13.9, 14–14.9, 15–15.9, 16–16.9, 17–17.9, and ≥18 mm. The follicle size was calculated by averaging the diameters of the follicle in two perpendicular planes.

Patient data were collected from our IVF electronic record systems, which contain comprehensive information on parental characteristics, medical and reproductive history, infertility causes, FET-related variables, laboratory parameters, and pregnancy and delivery outcomes (Wei *et al.*, 2023). The follow-up system has been extensively stated in our previous publications (Chen *et al.*, 2023; Liao *et al.*, 2024). Briefly, the couples received telephone interviews during each stage of pregnancy until delivery. The interview questionnaire collected information on pregnancy complications, birth date and locality, gestational weeks, mode of delivery, birth weight, infant gender, and neonatal diseases. Any adverse outcomes were adjudicated by a dedicated research nurse. When attempts to contact couples failed, data collection was obtained through interviews with the community family planning service. All follow-up information was maintained in our electronic medical database.

### Endometrial preparation protocols

In modified NC-FET, transvaginal ultrasound monitoring and serum hormone measurement were initiated from Day 10 of the menstrual cycle and subsequently repeated daily or every other day, depending on the size of the follicles and the serum hormone levels. The trigger time was primarily based on the LH and progesterone (P4) values (Zhang *et al.*, 2019b) and not on follicle size. Specifically, if the LH level was <20 IU/L, and the P4 level was <1.0 ng/ml, a bolus of urinary hCG (5000 IU; Lizhu Pharmaceuticals, Zhuhai, China) was administered at 21:00 to trigger ovulation, with embryo transfer scheduled either 5 days later for Day-3 embryos or 7 days later for blastocysts. Luteal phase support (LPS) commenced on the third day following hCG administration and consisted of vaginal progesterone (400 mg/day; Utrogestan; Besins, Paris, France) in combination with oral dydrogesterone (40 mg/day; Duphaston; Abbott, Hoofddorp, Netherlands) (Vuong *et al.*, 2021). If the LH level was ≥20 IU/l or the P_4_ level was ≥1.0 ng/ml, hCG was given immediately, followed by embryo transfers arranged 4 days later for Day-3 embryos and 6 days later for blastocysts. Luteal supplementation (Utrogestan plus dydrogesterone) began on the second day after hCG administration. Once pregnancy was confirmed, LPS continued until at least 8 weeks of gestation.

It should be mentioned that our center has adopted a 7-day work schedule, allowing for cycle monitoring and embryo transfer to be conducted on any day of the week. Furthermore, hCG could be administrated at night because as a university-affiliated IVF center, we offer injection services available 24 h a day.

### Embryo quality assessment and vitrification

Embryo morphology assessments were conducted on Days 3, 5, and 6. A good-quality embryo on Day 3 was classified as having at least six blastomeres and <20% fragmentation, in accordance with the Istanbul Consensus Workshop guidelines (Alpha Scientists in Reproductive Medicine and ESHRE Special Interest Group of Embryology, 2011). For Day 5 or 6 blastocysts, a quality grade of ≥3 BB was deemed indicative of good quality, based on the Gardner criteria (Gardner and Schoolcraft, 1999). Noticeably, the embryo transfer policy underwent a change in 2019 to align with Chinese legislation (Chen *et al.*, 2023). Prior to 2019, a maximum of two embryos could be transferred (Chen *et al.*, 2016; Shi *et al.*, 2018), whereas from 2019 onwards, a single-embryo transfer policy was implemented, particularly for women undergoing their first treatment cycles.

The vitrification and warming procedures were previously detailed by Kuwayama *et al.* (2005). Briefly, vitrification was performed using a Cryotop carrier system (Suyun Medical Materials Co., Jiangsu, China) with dimethyl sulfoxide–ethylene glycol–sucrose as cryoprotectants. For warming, the Cryotop strip was removed from the liquid nitrogen, and the embryos were transferred into a thawing solution sequentially (1 mol/l to 0.5 mol/l to 0 mol/l sucrose). The cryopreservation approach remained consistent during the study period (Liao *et al.*, 2024).

### Definitions and outcome measures

The primary outcome of interest was the live birth rate (LBR), defined as the delivery of a live baby after 24 weeks of gestation. Secondary outcomes were as follows: a positive pregnancy test was defined as a serum hCG level exceeding 5 mIU/ml, measured 14 days after FET; biochemical pregnancy loss was defined as a spontaneous pregnancy demise based on decreasing serum hCG levels without an ultrasound evaluation (Kolte *et al.*, 2015); the implantation rate was calculated as the number of gestational sacs per number of embryos transferred; a clinical pregnancy was confirmed through the visualization of at least one intrauterine gestational sac via ultrasound 4–5 weeks post-FET; and clinical pregnancy loss was defined as spontaneous clinical pregnancy loss prior to 24 weeks of gestation. Total pregnancy loss included both biochemical pregnancy loss and clinical pregnancy loss. Perinatal outcomes examined included the newborn’s gender, gestational age, and mean birthweight, as well as preterm birth (PTB, <37 gestational weeks), low birthweight (LBW, <2500 g), macrosomia (≥4000 g), small-for-gestational-age (SGA, birthweight <10th percentile, adjusted for gestational age and newborn gender using the updated nationwide birthweight curve in China) (Miao *et al.*, 2019; Huang *et al.*, 2022), and large-for-gestational-age (LGA, birthweight >90th percentile, similarly adjusted). The obstetrical complications were defined using the International Statistical Classification of Diseases Tenth Revision (ICD-10) codes. The analyzed outcomes were hypertensive disorder of pregnancy (HDP) (ICD-10: O13–O15), gestational diabetes mellitus (GDM) (ICD-10: O24.4), preterm premature rupture of membranes (PPROM) (ICD-10: O42), abnormal placentation (including placental previa [ICD-10: O44] and placental abruption [ICD-10: O45]), and mode of delivery. Only live birth deliveries were analyzed in relation to perinatal outcomes and maternal complications.

### Statistical analyses

All data were processed using SPSS (version 26.0; IBM, Armonk, NY, USA). Continuous variables were expressed as mean± SD and analyzed by one-way analysis of variance. Categorical variables were described as numbers (n) and percentages (%) and were compared using the chi-square or Fisher’s exact test, as appropriate. A *post-hoc* Bonferroni correction was applied for multiple comparisons. Logistic regression employing a generalized estimating equation was utilized to adjust for potentially relevant confounders, while also accounting for multiple cycles per patient. The adjusted models included the following variables: maternal age and BMI, parity, infertility duration and cause, insemination method, the number of oocytes retrieved, rank of FET, endometrial thickness, number of embryos transferred, embryo stage at transfer, embryo quality, levels of estradiol (E2), LH, and P4 on the hCG trigger day, E2 and P4 levels on the day of FET, and year of treatment. Based on protocols employed in IVF ovarian stimulation cycles aimed at obtaining mature oocytes (where hCG is administered when two or more follicles reach a diameter of ≥18 mm), the reference group has been defined as follicle size ≥18 mm in the regression models. A *P*-value <0.05 was considered as statistically significant.

Additionally, four sensitivity analyses were conducted to check the robustness of the primary findings. The first was restricted to women who underwent the first FET cycle. The second analysis focused on nulliparous women because a prior delivery may affect the subsequent birth and maternal outcomes. The third one was based on the outcomes of twin deliveries. Finally, a sensitivity analysis with follicle size as a continuous variable (including both very small and large follicles) was undertaken.

## Results

A total of 14 970 modified NC-FET cycles were recorded in the database during the study period, of which 14 431 that met the inclusion criteria were included for final analysis. Supplementary Fig. S1 illustrates a flow chart of the study population. Demographic background data and main cycle characteristics, categorized by dominant follicle size, are detailed in Table 1. There was no significant difference among the eight follicle-size groups in terms of BMI, parity, infertility cause, number of embryos transferred, embryo developmental stage at transfer, or the transfer of good-quality embryos. Conversely, maternal age, infertility duration, fertilization method, FET cycle rank, LH levels on the trigger day, the number of oocytes retrieved, and the year of treatment differed significantly among the groups. Furthermore, E2 levels, measured either on the day of the hCG trigger or on the day of embryo transfer, progressively increased, corresponding to the increasing follicle size (both *P* < 0.001). Additionally, the P4 levels on the day of triggering varied significantly across the cohorts (*P* < 0.001): the highest levels were observed in the group with follicle size <12 mm (1.11 ± 0.73 ng/ml), followed by those with follicle sizes between 12 and 12.9 mm (0.78 ± 0.53 ng/ml) and between 13 and 13.9 mm (0.62 ± 0.42 ng/ml). Furthermore, it was noted that the endometrial thickness was lowest (9.91 ± 2.46 mm) in the group with the smallest follicle size (<12 mm), whilst the highest endometrial thickness (10.57 ± 2.14 mm) was seen in the largest follicle size group (≥18 mm) (*P* < 0.001).

**Table 1.** Baseline and treatment characteristics of FET cycles, according to dominant follicle size.

	<12 mm	12–12.9 mm	13–13.9 mm	14–14.9 mm	15–15.9 mm	16–16.9 mm	17–17.9 mm	≥18 mm	*P* value
**Cycles (n)**	102	305	486	908	1378	2053	2515	6684	
**Mean diameter (mm)**	11.05 ± 0.47	12.42 ± 0.28	13.47 ± 0.30	14.48 ± 0.28	15.48 ± 0.29	16.45 ± 0.29	17.43 ± 0.28	19.86 ± 1.73	<0.001*
**Age (years)**	34.27 ± 4.82	34.25 ± 5.11	33.96 ± 5.17	34.51 ± 5.06	34.07 ± 5.02	33.89 ± 4.89	33.80 ± 4.80	33.64 ± 4.85	<0.001*
**BMI (kg/m^2^)**	21.56 ± 2.56	21.55 ± 2.95	21.45 ± 2.95	21.51 ± 2.83	21.33 ± 2.82	21.29 ± 2.72	21.40 ± 2.75	21.42 ± 2.79	0.422*
**Infertility duration (years)**	3.60 ± 3.05	3.64 ± 2.85	3.52 ± 3.16	3.91 ± 3.29	3.65 ± 3.32	3.64 ± 3.15	3.53 ± 3.10	3.48 ± 3.04	0.008*
**Parity, n (%)**									0.252**
0	85 (83.3)	267 (87.5)	431 (88.7)	772 (85.0)	1187 (86.1)	1807 (88.0)	2183 (86.8)	5835 (87.3)	
≥1	17 (16.7)	38 (12.5)	55 (11.3)	136 (15.0)	191 (13.9)	246 (12.0)	332 (13.2)	849 (12.7)	
**Main infertility cause, n (%)**									0.582**
Female	51 (50.0)	178 (58.4)	265 (54.5)	531 (58.5)	783 (56.8)	1166 (56.8)	1417 (56.3)	3831 (57.3)	
Male	20 (19.6)	43 (14.1)	64 (13.2)	118 (13.0)	175 (12.7)	263 (12.8)	339 (13.5)	820 (12.3)	
Mixed	23 (22.5)	70 (23.0)	136 (28.0)	211 (23.2)	336 (24.4)	490 (23.9)	598 (23.8)	1635 (24.5)	
Unexplained	8 (7.8)	14 (4.6)	21 (4.3)	48 (5.3)	84 (6.1)	134 (6.5)	161 (6.4)	398 (6.0)	
**Fertilization method, n (%)**									0.006**
IVF	59 (57.8)	193 (63.3)	323 (66.5)	589 (64.9)	869 (63.1)	1264 (61.6)	1523 (60.6)	4184 (62.6)	
ICSI	30 (29.4)	95 (31.1)	133 (27.4)	254 (28.0)	403 (29.2)	628 (30.6)	731 (29.1)	1961 (29.3)	
IVF + ICSI	13 (12.7)	17 (5.6)	30 (6.2)	65 (7.2)	106 (7.7)	161 (7.8)	261 (10.4)	539 (8.1)	
**FET cycle rank, n (%)**									<0.001**
First	45 (44.1)	156 (51.1)	260 (53.5)	497 (54.7)	796 (57.8)	1161 (56.6)	1426 (56.7)	3499 (52.3)	
High order	57 (55.9)	149 (48.9)	226 (46.5)	411 (45.3)	582 (42.2)	892 (43.4)	1089 (43.3)	3185 (47.7)	
**Endometrial thickness (mm)**	9.91 ± 2.46	10.28 ± 2.07	10.37 ± 2.15	10.15 ± 2.09	10.14 ± 1.96	10.21 ± 1.97	10.36 ± 2.06	10.57 ± 2.14	<0.001*
**LH on the day of triggering (IU/l)**	15.29 ± 10.72	19.20 ± 13.19	21.46 ± 13.58	22.20 ± 14.99	24.45 ± 15.94	23.91 ± 15.19	25.51 ± 15.72	25.55 ± 15.76	<0.001*
<20	71 (69.6)	196 (64.3)	281 (57.8)	534 (58.8)	689 (50.0)	1076 (52.4)	1177 (46.8)	3023 (45.2)	<0.001**
≥20	31 (30.4)	109 (35.7)	205 (42.2)	374 (41.2)	689 (50.0)	977 (47.6)	1338 (53.2)	3661 (54.8)	<0.001**
**E2 on the day of triggering (pg/ml)**	117.56 ± 57.82	154.48 ± 68.01	180.34 ± 70.55	204.64 ± 82.16	223.29 ± 82.04	231.27 ± 86.00	243.99 ± 89.37	271.64 ± 105.25	<0.001*
**P4 on the day of triggering (ng/m)**	1.11 ± 0.73	0.78 ± 0.53	0.62 ± 0.42	0.53 ± 0.41	0.50 ± 0.34	0.52 ± 0.36	0.53 ± 0.33	0.61 ± 0.37	<0.001*
**E2 on the day of FET (pg/ml)**	97.32 ± 39.06	101.99 ± 40.60	107.74 ± 47.01	109.24 ± 51.61	109.60 ± 50.51	110.62 ± 49.61	113.95 ± 48.20	122.74 ± 52.76	<0.001*
**P4 on the day of FET (ng/m)**	17.06 ± 6.21	18.06 ± 5.30	17.72 ± 5.44	17.16 ± 5.67	17.18 ± 5.73	17.80 ± 6.06	17.69 ± 5.95	18.36 ± 6.48	<0.001*
**Number of oocytes retrieved**	9.42 ± 6.14	9.12 ± 6.43	8.99 ± 6.47	8.99 ± 6.36	9.35 ± 6.73	9.56 ± 6.72	9.72 ± 6.63	9.60 ± 6.49	0.033*
**Number of embryos transferred, n (%)**									0.094**
1	30 (29.4)	65 (21.3)	115 (23.7)	218 (24.0)	334 (24.2)	508 (24.7)	551 (21.9)	1663 (24.9)	
2	72 (70.6)	240 (78.7)	371 (76.3)	690 (76.0)	1044 (75.8)	1545 (75.3)	1964 (78.1)	5021 (75.1)	
**Embryo developmental stage, n (%)**									0.288**
Day 3	92 (90.2)	262 (85.9)	426 (87.7)	789 (86.9)	1188 (86.2)	1767 (86.1)	2154 (85.6)	5671 (84.8)	
Day 5/6	10 (9.8)	43 (14.1)	60 (12.3)	119 (13.1)	190 (13.8)	286 (13.9)	361 (14.4)	1013 (15.2)	
**Embryo quality at transfer of best embryo transferred, n (%)**									0.651**
Grade 1	99 (97.1)	286 (93.8)	454 (93.4)	846 (93.2)	1300 (94.3)	1933 (94.2)	2355 (93.6)	6243 (93.4)	
Grade 2	3 (2.9)	19 (6.2)	32 (6.6)	62 (6.8)	78 (5.7)	120 (5.8)	160 (6.4)	441 (6.6)	
**Year of treatment, n (%)**									<0.001**
2013–2015	33 (32.4)	166 (54.4)	242 (49.8)	463 (51.0)	708 (51.4)	1035 (50.4)	1251 (49.7)	3098 (46.3)	
2016–2019	60 (58.8)	130 (42.6)	213 (43.8)	343 (37.8)	530 (38.5)	794 (38.7)	1017 (40.4)	2892 (43.3)	
2020–2023	9 (8.8)	9 (3.0)	31 (6.4)	102 (11.2)	140 (10.2)	224 (10.9)	247 (9.8)	694 (10.4)	

Data are presented as mean± SD or n (%).

FET, frozen embryo transfer; E2, estradiol; P4, progesterone.

* One-way ANOVA.

** *χ* ^2^ test.

The pregnancy results are displayed in Table 2. The likelihood of live birth did not show a significant difference among the eight groups (*P* = 0.405), with LBRs of 37.3, 36.4, 36.0, 35.7, 37.2, 37.6, 37.2, and 39.0% for follicle sizes at trigger ranging from <12, 12–12.9, 13–13.9, 14–14.9, 15–15.9, 16–16.9, 17–17.9, and ≥18 mm, respectively. Additionally, there were no statistical differences with regard to the rates of positive pregnancy test (*P* = 0.541), biochemical pregnancy loss (*P* = 0.983), implantation (*P* = 0.230), clinical pregnancy (*P* = 0.631), clinical pregnancy loss (*P* = 0.713), and total pregnancy loss (*P* = 0.924), across the various follicle size ranges. Bonferroni testing also did not reveal any differences in the aforementioned reproductive parameters. In the unadjusted analyses, relative to the reference group, triggering at smaller follicle sizes (from <12 to 17.9 mm) did not compromise the odds of a positive pregnancy test, biochemical pregnancy loss, implantation, clinical pregnancy, pregnancy loss, or live birth. Furthermore, multivariable regression analyses indicated that, after adjusting for several key confounders, no significant differences were observed in any of the pregnancy outcomes when comparing the reference group to the other follicle size categories. Importantly, a subgroup analysis concentrating on the first treatment cycle confirmed the main findings (Supplementary Tables S1 and S2). Moreover, when treating follicle size as a continuous variable, there remained no significant association between the follicle size and any reproductive outcomes (Supplementary Table S3).

**Table 2.** Main reproductive outcomes of the FET cycles, according to dominant follicle size.

Group	<12 mm	12–12.9 mm	13–13.9 mm	14–14.9 mm	15–15.9 mm	16–16.9 mm	17–17.9 mm	≥18 mm	*P* value
**Cycles (n)**	102	305	486	908	1378	2053	2515	6684	
**Positive pregnancy test**	50/102 (49.0)^a^	149/305 (48.9)^a^	231/486 (47.5)^a^	437/908 (48.1)^a^	674/1378 (48.9)^a^	1002/2053 (48.8)^a^	1231/2515 (48.9)^a^	3387/6684 (50.7)^a^	0.541*
OR (95% CI)	0.94 (0.63–1.38)	0.93 (0.74–1.17)	0.88 (0.73–1.06)	0.90 (0.79–1.04)	0.93 (0.83–1.05)	0.93 (0.84–1.03)	0.93 (0.85–1.02）	Ref	
Adjusted OR (95% CI)**	1.15 (0.76–1.72)	1.02 (0.81–1.30)	0.93 (0.76–1.13)	0.99 (0.85–1.14)	0.97 (0.86–1.09)	0.95 (0.85–1.06)	0.92 (0.83–1.01)	Ref	
**Implantation**	58/174 (33.3)^a^	155/545 (28.4)^a^	258/857 (30.1)^a^	481/1598 (30.1)^a^	772/2422 (31.9)^a^	1128/3598 (31.4)^a^	1387/4479 (31.0)^a^	3786/11705 (32.3)^a^	0.230*
OR (95% CI)	1.05 (0.76–1.44)	0.83 (0.69–1.01)	0.90 (0.78–1.05)	0.90 (0.80–1.01)	0.98 (0.89–1.08)	0.96 (0.88–1.04)	0.94 (0.87–1.01)	Ref	
Adjusted OR (95% CI)**	1.23 (0.86–1.75)	0.92 (0.76–1.13)	0.95 (0.80–1.12)	0.98 (0.86–1.11)	1.01 (0.90–1.12)	0.97 (0.88–1.06)	0.94 (0.86–1.02)	Ref	
**Clinical pregnancy**	46/102 (45.1)^a^	132/305 (43.3)^a^	209/486 (43.0)^a^	396/908 (43.6)^a^	606/1378 (44.0)^a^	903/2053 (44.0)^a^	1099/2515 (43.7)^a^	3049/6684 (45.6)^a^	0.631*
OR (95% CI)	0.98 (0.66–1.45)	0.91 (0.72–1.15)	0.90 (0.75–1.08)	0.92 (0.80–1.06)	0.94 (0.83–1.05)	0.94 (0.85–1.03)	0.93 (0.84–1.02)	Ref	
Adjusted OR (95% CI)**	1.20 (0.79–1.80)	0.99 (0.78–1.25)	0.94 (0.78–1.15)	1.01 (0.87–1.17)	0.97 (0.86–1.10)	0.96 (0.86–1.07)	0.91 (0.83–1.01)	Ref	
**Total pregnancy loss**	12/50 (24.0)^a^	38/149 (25.5)^a^	56/231 (24.2)^a^	113/437 (25.9)^a^	161/674 (23.9)^a^	230/1002 (23.0)^a^	295/1231 (24.0)^a^	780/3387 (23.0)^a^	0.924*
OR (95% CI)	1.06 (0.55–2.03)	1.14 (0.79–1.67)	1.07 (0.78–1.46)	1.17 (0.93–1.47)	1.05 (0.86–1.27)	0.99 (0.84–1.18)	1.05 (0.90–1.23)	Ref	
Adjusted OR (95% CI)**	0.97 (0.50–1.87)	1.12 (0.76–1.63)	1.07 (0.77–1.48)	1.11 (0.87–1.40)	1.01 (0.82–1.25)	0.97 (0.81–1.15)	1.03 (0.88–1.21)	Ref	
**Biochemical pregnancy loss**	4/50 (8.0)^a^	17/149 (11.4)^a^	22/231 (9.5)^a^	41/437 (9.4)^a^	68/674 (10.1)^a^	99/1002 (9.9)^a^	132/1231 (10.7)^a^	338/3387 (10.0)^a^	0.983*
OR (95% CI)	0.78 (0.28–2.19)	1.16 (0.69–1.95)	0.95 (0.60–1.49)	0.93 (0.66–1.31)	1.01 (0.77–1.33)	0.99 (0.78–1.25)	1.08 (0.88–1.34)	Ref	
Adjusted OR (95% CI)**	0.73 (0.26–2.08)	1.17 (0.70–1.97)	0.96 (0.60–1.53)	0.90 (0.64–1.28)	1.00 (0.75–1.33)	0.96 (0.76–1.22)	1.07 (0.87–1.33)	Ref	
**Clinical pregnancy loss**	8/46 (17.4)	21/132 (15.9)^a^	34/209 (16.3)^a^	72/396 (18.2)^a^	93/606 (15.3)^a^	131/903 (14.5)^a^	163/1099 (14.8)^a^	442/3049 (14.5)^a^	0.713*
OR (95% CI)	1.24 (0.58–2.68)	1.12 (0.69–1.80)	1.15 (0.78–1.68)	1.31 (0.99–1.73)	1.07 (0.84–1.36)	1.00 (0.81–1.24)	1.03 (0.85–1.25)	Ref	
Adjusted OR (95% CI)**	1.14 (0.52–2.51)	1.10 (0.67–1.79)	1.13 (0.76–1.67)	1.27 (0.95–1.69)	1.01 (0.78–1.31)	0.98 (0.79–1.22)	1.00 (0.81–1.23)	Ref	
**Live birth**	38/102 (37.3)	111/305 (36.4)^a^	175/486 (36.0)^a^	324/908 (35.7)^a^	513/1378 (37.2)^a^	772/2053 (37.6)^a^	936/2515 (37.2)^a^	2607/6684 (39.0)^a^	0.405*
OR (95% CI)	0.93 (0.62–1.39)	0.90 (0.71–1.14)	0.88 (0.73–1.07)	0.87 (0.75–1.01)	0.93 (0.82–1.05)	0.94 (0.85–1.04）	0.93 (0.84–1.02)	Ref	
Adjusted OR (95% CI)**	1.13 (0.74–1.72)	0.98 (0.77–1.25)	0.92 (0.75–1.13)	0.96 (0.82–1.11)	0.97 (0.85–1.10)	0.97 (0.87–1.08)	0.92 (0.83–1.02)	Ref	

Data are presented as n (%).

OR, odds ratio.

* *χ* ^2^-test with Bonferroni adjustment; each superscript letter denotes a subset of Group categories whose column proportions do not differ significantly from each other at the 0.05 level.

** Analyses were adjusted for age, BMI, infertility duration, parity, main infertility cause, fertilization method, FET cycle rank, endometrial thickness, number of embryos transferred, embryo developmental stage, embryo quality, levels of E2, LH, and P4 on the trigger day, E2 and P4 levels on the day of FET, number of oocytes retrieved, and year of treatment.

Table 3 summarizes the obstetric complications of each study cohort. In comparison to the women in the reference group, pregnancies originating from the other study groups did not show elevated risks of GDM, HDP, abnormal placentation, PPROM, or cesarean section in the unadjusted analyses. Furthermore, even after adjusting for potential confounding variables, no associations were found between pregnancy complications and the varying follicle sizes at the trigger.

**Table 3.** Maternal complications of the singleton newborns, according to dominant follicle size.

Group	<12 mm	12–12.9 mm	13–13.9 mm	14–14.9 mm	15–15.9 mm	16–16.9 mm	17–17.9 mm	≥18 mm	*P*-value
	n = 30	n = 92	n = 136	n= 2 48	n = 378	n = 585	n = 705	n = 2030	
**Gestational diabetes mellitus**	1 (3.3)^a^	11 (12.0)^a^	7 (5.1)^a^	25 (10.1)^a^	38 (10.1)^a^	58 (9.9)^a^	51 (7.2)^a^	181 (8.9)^a^	0.267*
OR (95% CI)	0.35 (0.05–2.60)	1.39 (0.73–2.65)	0.55 (0.26–2.65)	1.15 (0.74–1.78)	1.14 (0.79–1.65)	1.12 (0.82–1.54)	0.80 (0.58–1.10)	Ref	
Adjusted OR (95% CI)**	0.39 (0.05–2.83)	1.29 (0.66–2.51)	0.55 (0.25–1.21)	1.14 (0.72–1.78)	1.05 (0.72–1.55)	1.13 (0.81–1.56)	0.77 (0.55–1.08)	Ref	
**Hypertensive disorders of pregnancy**	0 (0)^a^	4 (4.3)^a^	2 (1.5)^a^	9 (3.6)^a^	7 (1.9)^a^	6 (1.0)^a^	14 (2.0)^a^	37 (1.8)^a^	0.175*
OR (95% CI)	NA	2.45 (0.85–7.02)	0.80 (0.19–3.37)	2.03 (0.97–4.25)	1.02 (0.45–2.30)	0.56 (0.23–1.33)	1.09 (0.59–2.03)	Ref	
Adjusted OR (95% CI)**	NA	1.73 (0.55–5.52)	0.56 (0.12–2.70)	1.94 (0.92–4.07)	0.95 (0.41–2.24)	0.59 (0.24–1.47)	1.06 (0.56–2.03)	Ref	
**Abnormal placentation**	1 (3.3)^a^	4 (4.3)^a^	5 (3.7)^a^	6 (2.4)^a^	10 (2.6)^a^	17 (2.9)^a^	17 (2.4)^a^	36 (1.8)^a^	0.454*
OR (95% CI)	1.91 (0.25–14.41)	2.52 (0.88–7.23)	2.11 (0.82–5.48)	1.37 (0.57–3.29)	1.51 (0.74–3.06)	1.66 (0.92–2.97)	1.37 (0.76–2.45)	Ref	
Adjusted OR (95% CI)**	1.75 (0.21–14.89)	2.03 (0.70–5.93)	1.59 (0.62–4.11)	1.07 (0.44–2.60)	1.33 (0.64–2.75)	1.43 (0.78–2.64)	1.28 (0.70–2.34)	Ref	
**Preterm premature rupture of the membrane**	0 (0)^a^	3 (3.3)^a^	2 (1.5)^a^	7 (2.8)^a^	8 (2.1)^a^	8 (1.4)^a^	8 (1.1)^a^	32 (1.6)^a^	0.515*
OR (95% CI)	NA	2.11 (0.63–7.00)	0.93 (0.22–3.93)	1.81 (0.79–4.15)	1.35 (0.62–2.95)	0.87 (0.40–1.89)	0.72 (0.33–1.56)	Ref	
Adjusted OR (95% CI)**	NA	1.95 (0.53–7.15)	0.83 (0.18–3.87)	1.76 (0.73–4.25)	1.54 (0.69–3.42)	0.97 (0.42–2.21)	0.75 (0.33–1.72)	Ref	
**Caesarean section**	22 (73.3)^a^	63 (68.5)^a^	104 (76.5)^a^	168 (67.7)^a^	270 (71.4)^a^	380 (65.0)^a^	470 (66.7)^a^	1380 (68.0)^a^	0.189*
OR (95% CI)	1.30 (0.57–2.93)	1.02 (0.65–1.60)	1.47 (0.98–2.20)	0.99 (0.75–1.31)	1.18 (0.93–1.50)	0.87 (0.72–1.06)	0.94 (0.79–1.13)	Ref	
Adjusted OR (95% CI)***	1.33 (0.59–3.01)	0.92 (0.56–1.50)	1.41 (0.92–2.16)	0.95 (0.71–1.27)	1.15 (0.89–1.48)	0.87 (0.71–1.06)	0.91 (0.75–1.10)	Ref	

Data are presented as n (%).

OR, odds ratio.

Hypertensive disorders of pregnancy including pregnancy-induced hypertension and pre-eclampsia. Abnormal placentation, including placenta praevia and placental abruption.

* *χ* ^2^-test with Bonferroni adjustment; each superscript letter denotes a subset of Group categories whose column proportions do not differ significantly from each other at the 0.05 level.

** Analyses were adjusted for age, BMI, infertility duration, parity, main infertility cause, fertilization method, FET cycle rank, endometrial thickness, number of embryos transferred, embryo developmental stage, embryo quality, levels of E2, LH, and P4 on the trigger day, E2 and P4 levels on the day of FET, number of oocytes retrieved and year of treatment.

*** Analyses were adjusted for age, BMI, infertility duration, parity, main infertility cause, fertilization method, FET cycle rank, endometrial thickness, number of embryos transferred, embryo developmental stage, embryo quality, levels of E2, LH, and P4 on the trigger day, E2 and P4 levels on the day of FET, number of oocytes retrieved and year of treatment, gestational diabetes, hypertensive disorder of pregnancy, abnormal placentation, and preterm premature rupture of membranes.

As listed in Table 4, a total of 4204 live-born singletons were analyzed for birth outcomes. Both the crude and confounder-adjusted models revealed that neonates born to women who underwent ovulation triggering at smaller follicle sizes (<12–17.9 mm) exhibited comparable perinatal outcomes, including PTB, LBW, macrosomia, SGA, and LGA, when compared with those from the reference cohorts. Additionally, other parameters, such as gestational age, mean birth weight, and gender distribution, were found to be similar between the study and control groups. Moreover, maternal and birth outcomes of twin deliveries were also comparable between the reference group and the other follicle size categories (Supplementary Tables S4 and S5).

**Table 4.** Perinatal outcomes of singletons born after FET cycles, according to dominant follicle size.

Group	<12 mm	12–12.9 mm	13–13.9 mm	14–14.9 mm	15–15.9 mm	16–16.9 mm	17–17.9 mm	≥18 mm	*P*-value
	n = 30	n = 92	n = 136	n = 248	n = 378	n = 585	n = 705	n = 2030	
**Gestational age**	38.43 ± 2.37	38.42 ± 1.73	38.58 ± 1.57	38.50 ± 1.60	38.66 ± 1.43	38.70 ± 1.35	38.60 ± 1.47	38.57 ± 1.48	0.542*
**Birthweight (g)**	3344.17 ± 604.12	3320.43 ± 548.88	3399.07 ± 475.86	3300.52 ± 505.76	3398.51 ± 485.76	3318.95 ± 458.82	3332.67 ± 455.76	3357.77 ± 474.71	0.089*
**Sex**									0.985**
Female	15 (50.0)^a^	43 (46.7)^a^	62 (45.6)^a^	124 (50.0)^a^	188 (49.7)^a^	283 (48.4)^a^	337 (47.8)^a^	967 (47.6)^a^	
Male	15 (50.0)^a^	49 (53.3)^a^	74 (54.4)^a^	124 (50.0)^a^	190 (50.3)^a^	302 (51.6)^a^	368 (52.2)^a^	1063 (52.4)^a^	
**PTB**	2 (6.7)^a^	6 (6.5)^a^	7 (5.1)^a^	17 (6.9)^a^	24 (6.3)^a^	23 (3.9)^a^	32 (4.5)^a^	108 (5.3)^a^	0.607**
OR (95% CI)	1.27 (0.30–5.41)	1.24 (0.53–2.91)	0.97 (0.44–2.12)	1.31 (0.77–2.22)	1.21 (0.76–1.91)	0.73 (0.46–1.15)	0.85 (0.57–1.27)	Ref	
Adjusted OR (95% CI)***	2.05 (0.53–7.97)	0.60 (0.23–1.54)	0.79 (0.33–1.93)	0.84 (0.43–1.68)	0.97 (0.54–1.76)	0.63 (0.35–1.12)	0.77 (0.48–1.25)	Ref	
**LBW (<2500 g)**	2 (6.7)^a^	3 (3.3)^a^	6 (4.4)^a^	14 (5.6)^a^	13 (3.4)^a^	19 (3.2)^a^	25 (3.5)^a^	70 (3.4)^a^	0.727**
OR (95% CI)	2.00 (0.47–8.56)	0.94 (0.29–3.06)	1.29 (0.55–3.03)	1.68 (0.93–3.02)	0.99 (0.55–1.82)	0.94 (0.56–1.57)	1.03 (0.65–1.64)	Ref	
Adjusted OR (95% CI)***	3.26 (0.84–12.67)	0.83 (0.23–3.02)	1.27 (0.55–2.94)	1.30 (0.66–2.56)	0.88 (0.44–1.73)	0.97 (0.55–1.69)	0.99 (0.61–1.63)	Ref	
**Macrosomia (≥4000 g)**	4 (13.3)^a^	8 (8.7)^a^	12 (8.8)^a^	16 (6.5)^a^	38 (10.1)^a^	42 (7.2)^a^	56 (7.9)^a^	164 (8.1)^a^	0.687**
OR (95% CI)	1.75 (0.60–5.08)	1.08 (0.52–2.28)	1.10 (0.60–2.03)	0.79 (0.46–1.34)	1.27 (0.88–1.84)	0.88 (0.62–1.25)	0.98 (0.72–1.35)	Ref	
Adjusted OR (95% CI)***	1.90 (0.63–5.74)	0.93 (0.43–2.01)	0.98 (0.51–1.87)	0.79 (0.46–1.35)	1.20 (0.83–1.76)	0.90 (0.63–1.28)	0.95 (0.69–1.32)	Ref	
**SGA**	0 (0)^a^	6 (6.5)^a^	4 (2.9)^a^	12 (4.8)^a^	15 (4.0)^a^	32 (5.5)^a^	25 (3.5)^a^	91 (4.5)^a^	0.519**
OR (95% CI)	NA	1.49 (0.63–3.49)	0.65 (0.23–1.79)	1.08 (0.59–2.01)	0.88 (0.50–1.54)	1.23 (0.82–1.87)	0.78 (0.50–1.23)	Ref	
Adjusted OR (95% CI)***	NA	1.89 (0.81–4.40)	0.73 (0.23–2.27)	1.12 (0.58–2.17)	1.02 (0.56–1.83)	1.32 (0.86–2.03)	0.79 (0.49–1.26)	Ref	
**LGA**	6 (20.0)^a^	15 (16.3)^a^	21 (15.4)^a^	30 (12.1)^a^	67 (17.7)^a^	78 (13.3)^a^	99 (14.0)^a^	327 (16.1)^a^	0.342**
OR (95% CI)	1.30 (0.53–3.21)	1.02 (0.58–1.79)	0.95 (0.59–1.54)	0.72 (0.48–1.07)	1.12 (0.84–1.50)	0.80 (0.61–1.05)	0.85 (0.67–1.09)	Ref	
Adjusted OR (95% CI)***	1.42 (0.56–3.63)	0.86 (0.48–1.54)	0.85 (0.52–1.39)	0.71 (0.47–1.06)	1.05 (0.78–1.43)	0.80 (0.61–1.06)	0.81 (0.63–1.04)	Ref	

Data are presented as mean± SD or n (%).

OR, odds ratio; FET, frozen embryo transfer; PTB, preterm birth; LBW, low birthweight; SGA, small for gestational age; LGA, large for gestational age.

* One-way ANOVA.

** *χ* ^2^-test with Bonferroni adjustment; each superscript letter denotes a subset of Group categories whose column proportions do not differ significantly from each other at the 0.05 level.

*** Analyses were adjusted for age, BMI, infertility duration, parity, main infertility cause, fertilization method, FET cycle rank, endometrial thickness, number of embryos transferred, embryo developmental stage, embryo quality, levels of E2, LH, and P4 on the trigger day, E2 and P4 levels on the day of FET, number of oocytes retrieved and year of treatment, gestational diabetes, hypertensive disorder of pregnancy, abnormal placentation, and preterm premature rupture of membranes.

Of note, the differences in pregnancy, obstetric, and perinatal outcomes remained non-significant when the analyses were restricted to nulliparous women, when comparing the reference control group with other study follicle size groups (Supplementary Tables S6, S7, and S8). Moreover, the distribution of cases for each lead follicle size at trigger at 1 mm intervals (but not aggregating data for follicle sized ≥18 mm) by year is documented in Supplementary Table S9.

## Discussion

To our knowledge, this is the largest study to date providing a detailed report on the pregnancy and delivery outcomes related to dominant follicle size in modified NC-FET cycles. Our findings indicate that by providing an intensive LPS, hCG triggering in modified NC-FETs can be administered at various follicle sizes, ranging from above 10 to 18 mm or larger. Notably, triggering ovulation at smaller-than-usual follicle sizes was not associated with diminished LBR and negative perinatal and obstetric outcomes.

Limited research has been conducted on cycle outcomes in modified NC-FETs based on follicle size. Weiss *et al.* (2021) established a simple yet effective FET protocol, in which P4 supplementation commenced as soon as the leading follicle measured ≥12 mm in diameter and the endometrial thickness reached 7 mm, without the administration of hCG for ovulation trigger. The authors reported favorable pregnancy outcomes, with an LBR of 52.4%. Notably, this regimen provided more flexibility and freedom when planning the date of FET, thereby enabling the avoidance of weekend transfers. However, caution is warranted when interpreting these findings due to the very small sample size, which included only 42 women. Most critically, the lack of a comparison group limits the ability to draw definitive conclusions.

Mendes Godinho *et al.* proposed an alternative approach for FET endometrial preparation, termed natural proliferative phase FET (NPP-FET). In this protocol, exogenous progesterone is administered during the proliferative phase after confirming sufficient endometrial growth and the presence of a dominant follicle, regardless of its average size (Mendes Godinho *et al.*, 2024). This strategy effectively merges the benefits of both AC-FET (regarding flexibility and ease of patient monitoring and transfer scheduling) and NC-FET (regarding the presence of a corpus luteum (CL), which is important in maternal circulatory adaptation during pregnancy). Notably, the LBR was significantly higher in the NPP-FET group when compared to the AC-FET group (49.1% versus 38.4%, *P* < 0.01), while the miscarriage rate was lower in the NPP-FET group (19.7% versus 34.9%, *P* < 0.01). It should be noted that the study design, populations, and methodologies differed greatly between their research and ours: we solely focused on the autologous cycle, whereas Mendes Godinho *et al.* also incorporated donor cycles; we categorized subjects into several groups based on follicle size at the trigger, whilst they defined the dominant follicle as being ≥14 mm and did not perform further analyses stratified by follicle diameter.

A recent study conducted by Alonso-Mayo *et al.* (2024) reported comparable clinical outcomes when hCG was administered to patients with a mean follicle size ranging from 13 to 22 mm in modified NC-FETs. Given a follicle growth rate of 1–1.5 mm/day (Baerwald *et al.*, 2009), this approach allows for flexible scheduling of FETs within a timeframe of 5–7 days. Noticeably, the authors included both autologous and donor oocyte cycles, and triggers administered to smaller follicles (13–15.9 mm) were more frequently observed in donor recipients, potentially limiting the generalizability of their findings to the broader IVF population. Moreover, the authors categorized subjects into three groups (13.0–15.9, 16.0–18.9, and ≥19.0 mm), whereas our study categorized patients into eight groups, from follicle sizes of above 10 to 18 mm or larger.

The aforementioned studies support the notion that the presence of a mature oocyte is not essential for FET. However, due to the limited research in this area, hCG has historically been administered in modified NC-FETs when the leading follicle reaches an average diameter of 17–18 mm (Groenewoud *et al.*, 2013; Liu *et al.*, 2020; Mackens *et al.*, 2020). Awaiting the follicle to reach a mature size increases clinic visits, and laboratories must be prepared for embryo transfers on any day of the week. Our findings suggested that hCG could be flexibly given, with the lead follicle size being in the range of above 10 mm to ≥18 mm. This wide range not only simplifies patient monitoring but also offers an opportunity to balance the laboratories’ workload, thus reducing transfers on busy days or weekends. Furthermore, a more extended timeframe for scheduling FET allows women to plan their travel or work leave more effectively. Our data also serve as a valuable resource for counseling women with a history of premature ovulation, indicating that an earlier hCG trigger at a smaller follicle size in the subsequent cycle does not compromise the outcomes.

Literature regarding the association of the follicle size at trigger with obstetric and perinatal outcomes following modified NC-FETs is lacking. Our study is the first to explore this topic, and we found no significant differences in adverse birth and maternal outcomes between the reference and various follicle-sized groups. Theoretically, follicles larger than 10 mm can ovulate due to adequate LH/chorionic gonadotropin receptor expression (Jeppesen *et al.*, 2012). The subsequent CL produces not only E2 and P4 but also vasoactive substances, including relaxin and vascular endothelial growth factors that are important for placentation and maternal circulatory function during pregnancy (Conrad *et al.*, 2019; Singh *et al.*, 2020). However, whether the lead follicle size on the trigger day might affect the functions of the resulting CL still awaits further investigation.

The present study has limitations. First, the number of singletons in certain subgroups was small and lacked adequate power to detect differences; therefore, caution should be taken when interpreting the results. Further studies with larger sample sizes are necessary to confirm our findings. Second, our center implemented an intensive LPS regimen, consisting of 40 mg of dydrogesterone and 400 mg of micronized vaginal progesterone. This approach is not widely used in ovulatory FETs. The possibility exists that this intensive regimen may have contributed to the favorable outcomes observed, given that smaller follicles may not secrete the same levels of sex steroids as large follicles. Consequently, future multi-center studies are warranted to determine the generalizability of our findings across other clinics utilizing different LPS protocols. Third, the present is a retrospective analysis, which introduces inherent biases and confounders that we were unable to control. Moreover, the triggering time was based on LH surge/P4 measurements and not on follicle size; thus, further randomized controlled studies (RCTs) are needed to confirm our findings. Fourth, the majority of women received two cleavage-stage embryo transfers, which may not be representative of current clinical practice. However, this transfer policy was changed in 2019 and, from then onwards, a single-embryo-transfer policy was in practice for patients undergoing their first IVF cycle. Future studies restricted to single blastocyst transfers are needed to validate our findings. Finally, prior studies showed that when LH is peaking, an additional hCG trigger might be detrimental (Fatemi *et al.*, 2010). Noticeably, all women in that study had FET scheduled after an identical time interval, regardless of whether FET was performed after a spontaneous LH surge or after administration of hCG. This might result in embryo-endometrial asynchrony because ovulation may occur at a later stage after hCG administration as compared with that following the spontaneous LH surge (Kosmas *et al.*, 2007).

The main strength of the study lies in its large sample size. This is so far the largest series of modified NC-FETs to evaluate cycle outcomes about the dominant follicle size at the hCG trigger. Furthermore, we not only assessed reproductive outcomes but also examined perinatal and obstetric complications. Additionally, we employed a broader range of follicle size categories to enhance the precision of our outcome measurements. Finally, while our focus was on women with regular menstrual cycles, the implications of our findings may extend to women with irregular or anovulatory cycles when undergoing mildly stimulated FET cycles.

## Conclusions

The present study demonstrated that the lead follicle size on the day of the hCG trigger, ranging from over 10 to 18 mm or larger, was not linked to adverse reproductive, obstetric, or perinatal outcomes during modified NC-FET cycles, in which an intensive LPS was applied. Nevertheless, clinicians should continue to exercise caution before adopting this approach until further RCTs validate our findings.

## Supplementary Material

hoaf047_Supplementary_Data

## Data Availability

The data underlying this article will be shared on reasonable request to the corresponding author.

## References

[hoaf047-B1] Alonso-Mayo C , Kohls G, Santos-Ribeiro S, Soares SR, Garcia-Velasco JA. Modified natural cycle allows a window of 7 days for frozen embryo transfer planning. Reprod Biomed Online 2024;49:103774. 38609793 10.1016/j.rbmo.2023.103774

[hoaf047-B2] Alpha Scientists in Reproductive Medicine and ESHRE Special Interest Group of Embryology. The Istanbul consensus workshop on embryo assessment: proceedings of an expert meeting. Hum Reprod 2011;26:1270–1283. 21502182 10.1093/humrep/der037

[hoaf047-B3] Baerwald AR , Walker RA, Pierson RA. Growth rates of ovarian follicles during natural menstrual cycles, oral contraception cycles, and ovarian stimulation cycles. Fertil Steril 2009;91:440–449. 18249401 10.1016/j.fertnstert.2007.11.054

[hoaf047-B4] Busnelli A , Schirripa I, Fedele F, Bulfoni A, Levi-Setti PE. Obstetric and perinatal outcomes following programmed compared to natural frozen-thawed embryo transfer cycles: a systematic review and meta-analysis. Hum Reprod 2022;37:1619–1641. 35553678 10.1093/humrep/deac073

[hoaf047-B5] Centers for Disease Control and Prevention. 2020 Assisted Reproductive Technology Fertility Clinic and National Summary Report. US Dept of Health and Human Services; 2022. https://stacks.cdc.gov/view/cdc/148215/cdc_148215_DS1.pdf (30 August 2024, date last accessed).

[hoaf047-B6] Chen D , Xu Q, Mao X, Zhang J, Wu L. Reproductive history does not compromise subsequent live birth and perinatal outcome following in-vitro fertilization: analysis of 25 329 first frozen-thawed embryo transfer cycles without preimplantation genetic testing for aneuploidy. Ultrasound Obstet Gynecol 2023;62:430–438. 37058394 10.1002/uog.26220

[hoaf047-B7] Chen ZJ , Shi Y, Sun Y, Zhang B, Liang X, Cao Y, Yang J, Liu J, Wei D, Weng N et al Fresh versus frozen embryos for infertility in the polycystic ovary syndrome. N Engl J Med 2016;375:523–533. 27509101 10.1056/NEJMoa1513873

[hoaf047-B8] Conrad KP , Petersen JW, Chi YY, Zhai X, Li M, Chiu KH, Liu J, Lingis MD, Williams RS, Rhoton-Vlasak A et al Maternal cardiovascular dysregulation during early pregnancy after in vitro fertilization cycles in the absence of a corpus luteum. Hypertension 2019;74:705–715. 31352818 10.1161/HYPERTENSIONAHA.119.13015 PMC6687559

[hoaf047-B9] Dalsgaard TH , Hvas AM, Kirkegaard KS, Jensen MV, Knudsen UB. Impact of frozen thawed embryo transfer in hormone substituted cycles on thrombotic risk markers. Thromb Res 2022;209:23–32. 34847404 10.1016/j.thromres.2021.11.016

[hoaf047-B10] Erden M , Mumusoglu S, Polat M, Yarali Ozbek I, Esteves SC, Humaidan P, Yarali H. The LH surge and ovulation re-visited: a systematic review and meta-analysis and implications for true natural cycle frozen thawed embryo transfer. Hum Reprod Update 2022;28:717–732. 35258085 10.1093/humupd/dmac012

[hoaf047-B11] Fatemi HM , Kyrou D, Bourgain C, Van den Abbeel E, Griesinger G, Devroey P. Cryopreserved-thawed human embryo transfer: spontaneous natural cycle is superior to human chorionic gonadotropin-induced natural cycle. Fertil Steril 2010;94:2054–2058. 20097333 10.1016/j.fertnstert.2009.11.036

[hoaf047-B12] Gardner DK , Schoolcraft WB. Culture and transfer of human blastocysts. Curr Opin Obstet Gynecol 1999;11:307–311. 10369209 10.1097/00001703-199906000-00013

[hoaf047-B13] Ghobara T , Gelbaya TA, Ayeleke RO. Cycle regimens for endometrial preparation prior to frozen embryo transfer. Cochrane Database Syst Rev 2025;6:CD003414. 40458990 10.1002/14651858.CD003414.pub4 PMC12131296

[hoaf047-B14] Ginström Ernstad E , Wennerholm UB, Khatibi A, Petzold M, Bergh C. Neonatal and maternal outcome after frozen embryo transfer: Increased risks in programmed cycles. Am J Obstet Gynecol 2019;221:126.e1–e121-126. e118. 10.1016/j.ajog.2019.03.010 30910545

[hoaf047-B15] Groenewoud ER , Cantineau AE, Kollen BJ, Macklon NS, Cohlen BJ. What is the optimal means of preparing the endometrium in frozen-thawed embryo transfer cycles? A systematic review and meta-analysis. Hum Reprod Update 2013;19:458–470. 23820515 10.1093/humupd/dmt030

[hoaf047-B16] Groenewoud ER , Cohlen BJ, Al-Oraiby A, Brinkhuis EA, Broekmans FJ, de Bruin JP, van den Dool G, Fleisher K, Friederich J, Goddijn M et al A randomized controlled, non-inferiority trial of modified natural versus artificial cycle for cryo-thawed embryo transfer. Hum Reprod 2016;31:1483–1492. 27179265 10.1093/humrep/dew120 PMC5853593

[hoaf047-B17] Groenewoud ER , Cohlen BJ, Macklon NS. Programming the endometrium for deferred transfer of cryopreserved embryos: hormone replacement versus modified natural cycles. Fertil Steril 2018;109:768–774. 29778369 10.1016/j.fertnstert.2018.02.135

[hoaf047-B18] Groenewoud ER , Macklon NS, Cohlen BJ; ANTARCTICA Study Group. The effect of elevated progesterone levels before HCG triggering in modified natural cycle frozen-thawed embryo transfer cycles. Reprod Biomed Online 2017;34:546–554. 28319018 10.1016/j.rbmo.2017.02.008

[hoaf047-B19] Huang XY , Zhu YF, Liu HL, Wu GW, Liu CY, Zeng DY, He J, Shi QX, Xu J, Zhu B et al [Birth weight curves of singleton neonates with a gestational age of 24-42 weeks and their regional differences in 11 cities of China: an analysis of 93 720 cases]. Zhongguo Dang Dai Er Ke Za Zhi 2022;24:482–491. 35644187 10.7499/j.issn.1008-8830.2112032 PMC9154362

[hoaf047-B20] Jeppesen JV , Kristensen SG, Nielsen ME, Humaidan P, Dal Canto M, Fadini R, Schmidt KT, Ernst E, Yding Andersen C. LH-receptor gene expression in human granulosa and cumulus cells from antral and preovulatory follicles. J Clin Endocrinol Metab 2012;97:E1524–E1531. 22659248 10.1210/jc.2012-1427 PMC3410279

[hoaf047-B21] Kolte AM , Bernardi LA, Christiansen OB, Quenby S, Farquharson RG, Goddijn M, Stephenson MD; ESHRE Special Interest Group, Early Pregnancy. Terminology for pregnancy loss prior to viability: a consensus statement from the ESHRE early pregnancy special interest group. Hum Reprod 2015;30:495–498. 25376455 10.1093/humrep/deu299

[hoaf047-B22] Kosmas IP , Tatsioni A, Fatemi HM, Kolibianakis EM, Tournaye H, Devroey P. Human chorionic gonadotropin administration vs. luteinizing monitoring for intrauterine insemination timing, after administration of clomiphene citrate: a meta-analysis. Fertil Steril 2007;87:607–612. 17173907 10.1016/j.fertnstert.2006.10.003

[hoaf047-B23] Kuwayama M , Vajta G, Kato O, Leibo SP. Highly efficient vitrification method for cryopreservation of human oocytes. Reprod Biomed Online 2005;11:300–308. 16176668 10.1016/s1472-6483(10)60837-1

[hoaf047-B24] Lawrenz B , Coughlan C, Melado L, Fatemi HM. The ART of frozen embryo transfer: back to nature!. Gynecol Endocrinol 2020;36:479–483. 32188299 10.1080/09513590.2020.1740918

[hoaf047-B25] Lawrenz B , Kalafat E, Ata B, Melado L, Del Gallego R, Elkhatib I, Fatemi H. Do women with severely diminished ovarian reserve undergoing modified natural-cycle in-vitro fertilization benefit from earlier trigger at smaller follicle size? Ultrasound Obstet Gynecol 2024;64:245–252. 38348612 10.1002/uog.27611

[hoaf047-B26] Lawrenz B , Melado L, Fatemi HM. Frozen embryo transfers in a natural cycle: how to do it right. Curr Opin Obstet Gynecol 2023;35:224–229. 36924405 10.1097/GCO.0000000000000862

[hoaf047-B27] Liao M , Xu Q, Mao X, Zhang J, Wu L, Chen Q. Paternal age does not jeopardize the live birth rate and perinatal outcomes after in vitro fertilization: an analysis based on 56,113 frozen embryo transfer cycles. Am J Obstet Gynecol 2024;230:354.e351–354.e313. 10.1016/j.ajog.2023.11.1224 37952870

[hoaf047-B28] Liu X , Shi W, Shi J. Natural cycle frozen-thawed embryo transfer in young women with regular menstrual cycles increases the live-birth rates compared with hormone replacement treatment: a retrospective cohort study. Fertil Steril 2020;113:811–817. 32147171 10.1016/j.fertnstert.2019.11.023

[hoaf047-B29] Mackens S , Santos-Ribeiro S, van de Vijver A, Racca A, Van Landuyt L, Tournaye H, Blockeel C. Frozen embryo transfer: a review on the optimal endometrial preparation and timing. Hum Reprod 2017;32:2234–2242. 29025055 10.1093/humrep/dex285

[hoaf047-B30] Mackens S , Stubbe A, Santos-Ribeiro S, Van Landuyt L, Racca A, Roelens C, Camus M, De Vos M, van de Vijver A, Tournaye H et al To trigger or not to trigger ovulation in a natural cycle for frozen embryo transfer: a randomized controlled trial. Hum Reprod 2020;35:1073–1081. 32395750 10.1093/humrep/deaa026

[hoaf047-B31] Mendes Godinho C , Soares SR, Nunes SG, Martínez JMM, Santos-Ribeiro S. Natural proliferative phase frozen embryo transfer-a new approach which may facilitate scheduling without hindering pregnancy outcomes. Hum Reprod 2024;39:1089–1097. 38531673 10.1093/humrep/deae061

[hoaf047-B32] Miao H , Yao F, Wu Y, Zhang X, He R, Li B, Zhao Q. Birth weight percentiles by sex and gestational age for twins born in southern China. Sci Rep 2019;9:757. 30679504 10.1038/s41598-018-36758-6 PMC6345857

[hoaf047-B33] Montagut M , Santos-Ribeiro S, De Vos M, Polyzos NP, Drakopoulos P, Mackens S, van de Vijver A, van Landuyt L, Verheyen G, Tournaye H et al Frozen-thawed embryo transfers in natural cycles with spontaneous or induced ovulation: the search for the best protocol continues. Hum Reprod 2016;31:2803–2810. 27798046 10.1093/humrep/dew263

[hoaf047-B34] Shi Y , Sun Y, Hao C, Zhang H, Wei D, Zhang Y, Zhu Y, Deng X, Qi X, Li H et al Transfer of fresh versus frozen embryos in ovulatory women. N Engl J Med 2018;378:126–136. 29320646 10.1056/NEJMoa1705334

[hoaf047-B35] Singh B , Reschke L, Segars J, Baker VL. Frozen-thawed embryo transfer: the potential importance of the corpus luteum in preventing obstetrical complications. Fertil Steril 2020;113:252–257. 32106972 10.1016/j.fertnstert.2019.12.007 PMC7380557

[hoaf047-B36] Smeenk J , Wyns C, De Geyter C, Kupka M, Bergh C, Cuevas Saiz I, De Neubourg D, Rezabek K, Tandler-Schneider A, Rugescu I et al; European IVF Monitoring Consortium (EIM) for the European Society of Human Reproduction and Embryology (ESHRE). ART in Europe, 2019: results generated from European registries by ESHRE^†^. Hum Reprod 2023;38:2321–2338.37847771

[hoaf047-B37] von Versen-Höynck F , Griesinger G. Should any use of artificial cycle regimen for frozen-thawed embryo transfer in women capable of ovulation be abandoned: yes, but what’s next for FET cycle practice and research? Hum Reprod 2022;37:1697–1703. 35640158 10.1093/humrep/deac125

[hoaf047-B38] von Versen-Höynck F , Schaub AM, Chi YY, Chiu KH, Liu J, Lingis M, Stan Williams R, Rhoton-Vlasak A, Nichols WW, Fleischmann RR et al Increased preeclampsia risk and reduced aortic compliance with in vitro fertilization cycles in the absence of a corpus luteum. Hypertension 2019;73:640–649. 30636552 10.1161/HYPERTENSIONAHA.118.12043 PMC6434532

[hoaf047-B39] Vuong LN , Pham TD, Le KTQ, Ly TT, Le HL, Nguyen DTN, Ho VNA, Dang VQ, Phung TH, Norman RJ et al Micronized progesterone plus dydrogesterone versus micronized progesterone alone for luteal phase support in frozen-thawed cycles (MIDRONE): a prospective cohort study. Hum Reprod 2021;36:1821–1831. 33930124 10.1093/humrep/deab093

[hoaf047-B40] Wei M , Chen D, Feng G, Mao X, Wu L, Chai W, Zhang J. Association of infertility cause with perinatal outcomes in a freeze-all policy: an analysis including 10,151 singleton newborns. AJOG Glob Rep 2023;3:100098. 36438543 10.1016/j.xagr.2022.100098 PMC9684699

[hoaf047-B41] Weiss A , Baram S, Geslevich Y, Goldman S, Nothman S, Beck-Fruchter R. Should the modified natural cycle protocol for frozen embryo transfer be modified? A prospective case series proof of concept study. Eur J Obstet Gynecol Reprod Biol 2021;258:179–183. 33444812 10.1016/j.ejogrb.2021.01.004

[hoaf047-B42] Yang X , Zhang J, Wu J, Huang J, Chen Q, Lu X, Lyu Q, Kuang Y, Wang Y. Association between the number of oocytes retrieved and neonatal outcomes after freeze-all IVF cycles. Hum Reprod 2019;34:1937–1947. 31621863 10.1093/humrep/dez137

[hoaf047-B43] Zaat T , Zagers M, Mol F, Goddijn M, van Wely M, Mastenbroek S. Fresh versus frozen embryo transfers in assisted reproduction. Cochrane Database Syst Rev 2021;2:CD011184. 33539543 10.1002/14651858.CD011184.pub3 PMC8095009

[hoaf047-B44] Zhang J , Liu H, Mao X, Chen Q, Fan Y, Xiao Y, Wang Y, Kuang Y. Effect of body mass index on pregnancy outcomes in a freeze-all policy: an analysis of 22,043 first autologous frozen-thawed embryo transfer cycles in China. BMC Med 2019a;17:114. 31238940 10.1186/s12916-019-1354-1 PMC6593528

[hoaf047-B45] Zhang J , Liu H, Wang Y, Mao X, Chen Q, Fan Y, Xiao Y, Kuang Y. Letrozole use during frozen embryo transfer cycles in women with polycystic ovary syndrome. Fertil Steril 2019b;112:371–377. 31126712 10.1016/j.fertnstert.2019.04.014

