# Correction to: Pregnancy and perinatal outcomes after modified natural cycle-frozen embryo transfers according to size of the dominant follicle on the hCG trigger day

**DOI:** 10.1093/hropen/hoaf066

**Published:** 2025-11-05

This is a correction to Jie Zhang, Shuwen Qiu, Hongyuan Gao, Xiaoyan Mao, Yi Guo, Ling Wu, Pregnancy and perinatal outcomes after modified natural cycle-frozen embryo transfers according to size of the dominant follicle on the hCG trigger day, *Human Reproduction Open*, Volume 2025, Issue 3, hoaf047, https://doi.org/10.1093/hropen/hoaf047.

The authors of the above article would like to apologise for a typographical error in the dose of dydrogesterone used for intensive luteal phase support listed in the final sentence of the ‘PARTICIPANTS/MATERIALS, SETTING, METHODS’ subsection of the extended abstract and the seventh paragraph of the Discussion.

The regimen used for intensive luteal phase support is mistakenly stated as:

“400 mg of dydrogesterone and 400 mg of micronized vaginal progesterone”

It should be:

“40 mg of dydrogesterone and 400 mg of micronized vaginal progesterone”

The final sentence in the PARTICIPANTS/MATERIALS, SETTING, METHODS’ subsection in the extended abstract should read as:

An intensive LPS, consisting of 40 mg dydrogesterone plus 400 mg vaginal progesterone, was adopted.

The opening sentences of the seventh paragraph of the Discussion should read as:

The present study has limitations. First, the number of singletons in certain subgroups was small and lacked adequate power to detect differences; therefore, caution should be taken when interpreting the results. Further studies with larger sample sizes are necessary to confirm our findings. Second, our center implemented an intensive LPS regimen, consisting of 40 mg of dydrogesterone and 400 mg of micronized vaginal progesterone.

The authors would like to assure readers that these typographical errors do not affect any results, conclusions or other content of the article.

The electronic version of this article has been updated at https://doi.org/10.1093/hropen/hoaf047.

