# Supplementary material for: Pregnancy and perinatal outcomes after modified natural cycle-frozen embryo transfers according to size of the dominant follicle on the hCG trigger day
Source: Hum Reprod Open. 2025 Jul 16;2025(3):hoaf047. doi: 10.1093/hropen/hoaf047 (PMC12343029; doi:10.1093/hropen/hoaf047)
Supplement: hoaf047_Supplementary_Data [file hoaf047_supplementary_data.zip › HRO-24-0370-R3-SuppFigS1.docx]

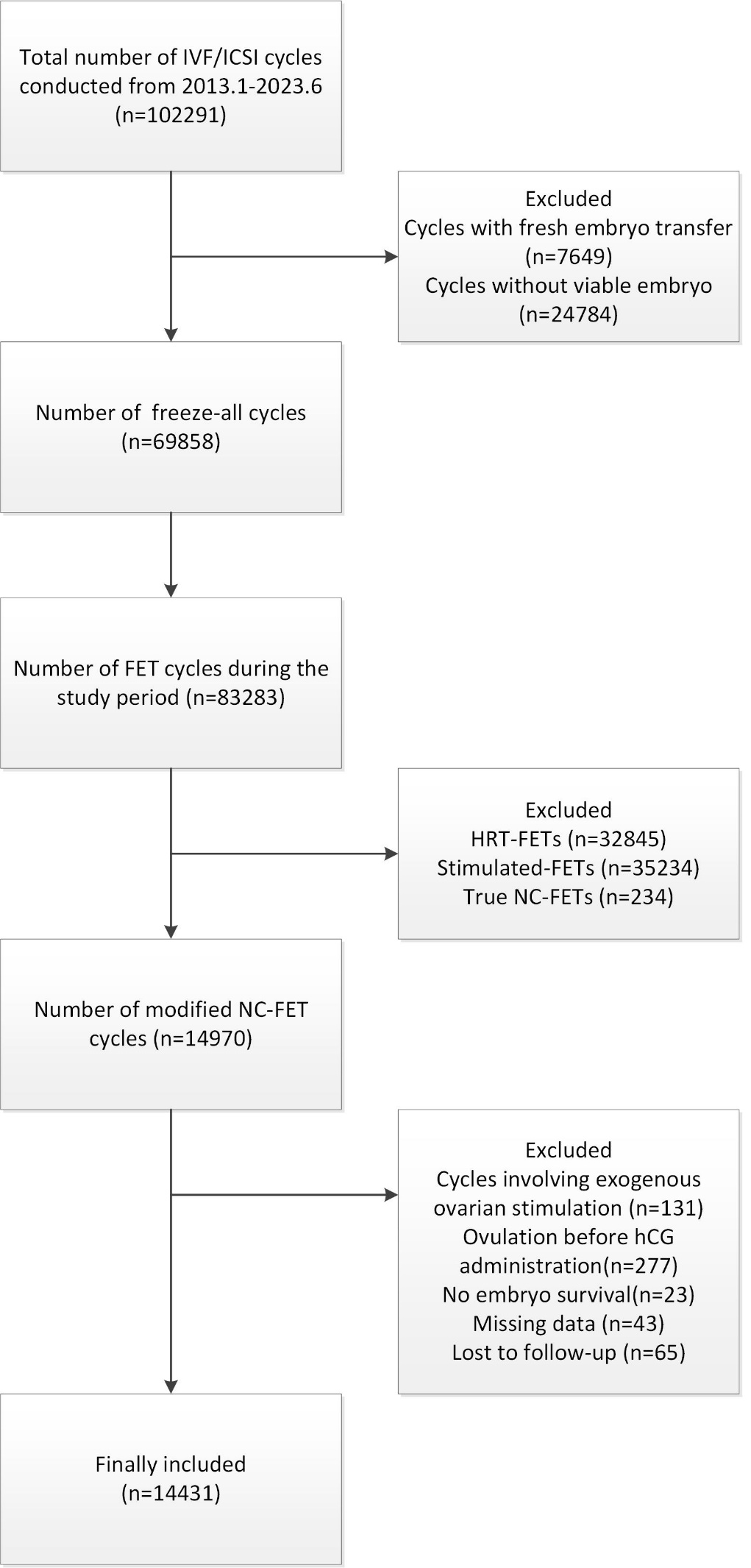


**Supplementary Figure S1. Flow chart illustrating the study population, including the inclusion and exclusion criteria.** IVF/ICSI and FET cycles that were performed between January 2013 and June 2023 were included for potential analysis. Cycles with fresh embryo transfers, cycles with no viable embryos to be transferred, other types of FETs, cycles lost to follow-up or with missing data, cycles with no embryo survival after thawing or cycles in which ovulation had occurred before hCG administration were all excluded. IVF, in vitro fertilization; ICSI, intracytoplasmic sperm injection; FET, frozen embryo transfer; NC, natural cycle; HRT, hormone replacement therapy.
