# Supplementary material for: Pregnancy and perinatal outcomes after modified natural cycle-frozen embryo transfers according to size of the dominant follicle on the hCG trigger day
Source: Hum Reprod Open. 2025 Jul 16;2025(3):hoaf047. doi: 10.1093/hropen/hoaf047 (PMC12343029; doi:10.1093/hropen/hoaf047)
Supplement: hoaf047_Supplementary_Data [file hoaf047_supplementary_data.zip › HRO-24-0370-R3-SuppTables1-9_EO.docx]

**Supplementary Table S1 Baseline and treatment characteristics of the first FET cycle, according to dominant follicle size.**

|  | **<12mm** | **12–12.9mm** | **13–13.9mm** | **14–14.9mm** | **15–15.9mm** | **16–16.9mm** | **17–17.9mm** | **≥18mm** | **P value** |
| --- | --- | --- | --- | --- | --- | --- | --- | --- | --- |
| **Cycles (n)** | 45 | 156 | 260 | 497 | 796 | 1161 | 1426 | 3499 |  |
| **Mean diameter (mm)** | 11.15±0.39 | 12.43±0.28 | 13.46±0.30 | 14.47±0.28 | 15.49±0.29 | 16.44±0.29 | 17.43±0.29 | 19.77±1.66 | <0.001^*^ |
| **Age (years)** | 33.44±5.36 | 33.56±4.91 | 33.28±5.06 | 33.49±4.80 | 33.44±4.82 | 33.04±4.69 | 33.01±4.68 | 32.96±4.68 | 0.066^*^ |
| **Body mass index (kg/m^2^)** | 21.17±2.66 | 21.57±2.85 | 21.56±2.97 | 21.58±2.91 | 21.40±2.81 | 21.23±2.64 | 21.45±2.75 | 21.45±2.84 | 0.269^*^ |
| **Infertility duration (years)** | 3.89±3.34 | 3.43±2.73 | 3.31±3.06 | 3.79±3.30 | 3.55±3.26 | 3.59±3.19 | 3.38±2.98 | 3.40±3.04 | 0.100^*^ |
| **Parity, n (%)** |  |  |  |  |  |  |  |  | 0.244^**^ |
| 0 | 37(82.2) | 133(85.3) | 230(88.5) | 418(84.1) | 685(86.1) | 1023(88.1) | 1250(87.7) | 3070(87.7) |  |
| ≥1 | 8(17.8) | 23(14.7) | 30(11.5) | 79(15.9) | 111(13.9) | 138(11.9) | 176(12.3) | 429(12.3) |  |
| **Main infertility cause, n (%)** |  |  |  |  |  |  |  |  | 0.480^**^ |
| Female | 26(57.8) | 92(59.0) | 142(54.6) | 280(56.3) | 424(53.3) | 630(54.3) | 781(54.8) | 1973(56.4) |  |
| Male | 10(22.2) | 19(12.2) | 35(13.5) | 73(14.7) | 112(14.1) | 161(13.9) | 211(14.8) | 432(12.3) |  |
| Mixed | 8(17.8) | 40(25.6) | 73(28.1) | 114(22.9) | 208(26.1) | 298(25.7) | 345(24.2) | 874(25.0) |  |
| Unexplained | 1(2.2) | 5(3.2) | 10(3.8) | 30(6.0) | 52(6.5) | 72(6.2) | 89(6.2) | 220(6.3) |  |
| **Fertilization method, n (%)** |  |  |  |  |  |  |  |  | 0.005^**^ |
| IVF | 29(64.4) | 101(64.7) | 175(67.3) | 329(66.2) | 492(61.8) | 692(59.6) | 844(59.2) | 2228(63.7) |  |
| ICSI | 12(26.7) | 46(29.5) | 69(26.5) | 131(26.4) | 225(28.3) | 364(31.4) | 413(29.0) | 932(26.6) |  |
| IVF+ICSI | 4(8.9) | 9(5.8) | 16(6.2) | 37(7.4) | 79(9.9) | 105(9.0) | 169(11.9) | 339(9.7) |  |
| **Endometrial thickness (mm)** | 9.59±2.56 | 10.26±2.12 | 10.28±2.12 | 10.30±2.14 | 10.15±1.93 | 10.23±1.99 | 10.43±2.10 | 10.64±2.15 | <0.001^*^ |
| **LH on the day of triggering (IU/L)** | 14.96±10.84 | 19.83±13.04 | 21.14±12.79 | 22.00±15.23 | 24.80±14.86 | 23.99±15.34 | 26.43±15.67 | 25.73±15.72 | <0.001^*^ |
| <20 | 32(71.1) | 98(62.8) | 147(56.5) | 298(60.0) | 380(47.7) | 612(52.7) | 628(44.0) | 1561(44.6) | <0.001^**^ |
| ≥20 | 13(28.9) | 58(37.2) | 113(43.5) | 199(40.0) | 416(52.3) | 549(47.3) | 798(56.0) | 1938(55.4) | <0.001^**^ |
| **E2 on the day of triggering (pg/ml)** | 111.56±54.85 | 155.33±66.05 | 174.81±67.68 | 206.78±81.94 | 223.31±81.11 | 231.96±85.43 | 241.90±87.96 | 270.27±102.07 | <0.001^*^ |
| **P4 on the day of triggering (ng/m)** | 1.16±0.72 | 0.75±0.48 | 0.63±0.43 | 0.51±0.41 | 0.50±0.33 | 0.51±0.35 | 0.53±0.31 | 0.60±0.36 | <0.001^*^ |
| **E2 on the day of FET (pg/ml)** | 93.26±39.64 | 100.63±38.77 | 103.97±41.79 | 107.24±50.03 | 105.84±47.07 | 106.99±48.13 | 111.36±47.25 | 119.50±50.83 | <0.001^*^ |
| **P4 on the day of triggering (ng/m)** | 17.90±6.36 | 17.91±5.35 | 17.66±5.25 | 17.12±5.54 | 17.12±5.61 | 17.69±6.12 | 17.70±5.84 | 18.24±6.25 | <0.001^*^ |
| **Number of oocytes retrieved** | 8.82±6.65 | 9.40±6.63 | 9.05±6.68 | 8.82±6.24 | 9.00±6.39 | 9.26±6.43 | 9.28±6.58 | 9.14±6.18 | 0.874^*^ |
| **Number of embryos transferred, n (%)** |  |  |  |  |  |  |  |  | 0.118^**^ |
| 1 | 11(24.4) | 24(15.4) | 59(22.7) | 116(23.3) | 168(21.1) | 249(21.4) | 285(20.0) | 811(23.2) |  |
| 2 | 34(75.6) | 132(84.6) | 201(77.3) | 381(76.7) | 628(78.9) | 912(78.6) | 1141(80.0) | 2688(76.8) |  |
| **Embryo developmental stage****, n (%)** |  |  |  |  |  |  |  |  | 0.199^**^ |
| Day 3 | 42(93.3) | 147(94.2) | 233(89.6) | 440(88.5) | 724(91.0) | 1055(90.9) | 1286(90.2) | 3116(89.1) |  |
| Day 5/6 | 3(6.7) | 9(5.8) | 27(10.4) | 57(11.5) | 72(9.0) | 106(9.1) | 140(9.8) | 383(10.9) |  |
| **Embryo quality at transfer of best embryo transferred, n (%)** |  |  |  |  |  |  |  |  | 0.190^**^ |
| Grade 1 | 44(97.8) | 150(96.2) | 247(95.0) | 466(93.8) | 772(97.0) | 1108(95.4) | 1350(94.7) | 3325(95.0) |  |
| Grade 2 | 1(2.2) | 6(3.8) | 13(5.0) | 31(6.2) | 24(3.0) | 53(4.6) | 76(5.3) | 174(5.0) |  |
| **Year of treatment, n (%)** |  |  |  |  |  |  |  |  | <0.001^**^ |
| 2013**–**2015 | 13(28.9) | 86(55.1) | 132(50.8) | 270(54.3) | 433(54.4) | 598(51.5) | 755(52.9) | 1695(48.4) |  |
| 2016**–**2019 | 30(66.7) | 64(41.0) | 110(42.3) | 173(34.8) | 291(36.6) | 454(39.1) | 544(38.1) | 1472(42.1) |  |
| 2020**–**2023 | 2(4.4) | 6(3.8) | 18(6.9) | 54(10.9) | 72(9.0) | 109(9.4) | 127(8.9) | 332(9.5) |  |

Data are presented as mean± SD or n (%).

FET, frozen embryo transfer; LH, luteinizing hormone; E2, estradiol; P4, progesterone.

* One-way ANOVA.

** χ2 test.

**Supplementary Table S2. Main reproductive outcomes** **of the first FET cycle, according to dominant follicle size.**

| **Group** | **<12mm** | **12–12.9mm** | **13–13.9mm** | **14–14.9mm** | **15–15.9mm** | **16–16.9mm** | **17–17.9mm** | **≥18mm** | **P value** |
| --- | --- | --- | --- | --- | --- | --- | --- | --- | --- |
| **Cycles (n)** | 45 | 156 | 260 | 497 | 796 | 1161 | 1426 | 3499 |  |
| **Positive pregnancy test** | 25/45(55.6) ^a^ | 88/156(56.4) ^a^ | 132/260(50.8) ^a^ | 255/497(51.3) ^a^ | 425/796(53.4) ^a^ | 598/1161(51.5) ^a^ | 734/1426(51.5) ^a^ | 1903/3499(54.4) ^a^ | 0.424^*^ |
| OR (95% CI) | 1.05(0.58-1.89) | 1.09(0.79-1.50) | 0.87(0.67-1.11) | 0.88(0.73-1.07) | 0.96(0.82-1.12) | 0.89(0.78-1.02) | 0.89(0.79-1.01） | Ref |  |
| adjusted OR (95% CI) ^**^ | 1.29(0.70-2.37) | 1.21(0.87-1.70) | 0.92(0.71-1.20) | 0.96(0.79-1.16) | 1.01(0.86-1.19) | 0.92(0.80-1.06) | 0.90(0.78-1.01) | Ref |  |
| **Implantation** | 31/79(39.2) ^a^ | 88/288(30.4) ^a^ | 150/461(32.5) ^a^ | 282/878(32.1) ^a^ | 503/1424(35.3) ^a^ | 699/2073(33.7) ^a^ | 843/2567(32.8) ^a^ | 2170/6187(35.1) ^a^ | 0.185^*^ |
| OR (95% CI) | 1.20(0.76-1.88) | 0.82(0.63-1.05) | 0.89(0.73-1.09) | 0.88(0.75-1.02) | 1.01(0.90-1.14) | 0.94(0.85-1.05) | 0.91(0.82-1.00) | Ref |  |
| adjusted OR (95% CI) ^**^ | 1.43(0.86-2.40) | 0.95(0.73-1.23) | 0.96(0.76-1.20) | 0.94(0.80-1.10) | 1.07(0.93-1.22) | 0.98(0.87-1.10) | 0.92(0.82-1.03) | Ref |  |
| **Clinical pregnancy** | 23/45(51.1) ^a^ | 76/156(48.7) ^a^ | 118/260(45.4) ^a^ | 230/497(46.3) ^a^ | 389/796(48.9) ^a^ | 552/1161(47.5) ^a^ | 660/1426(46.3) ^a^ | 1723/3499(49.2) ^a^ | 0.580^*^ |
| OR (95% CI) | 1.08(0.60-1.94) | 0.98(0.71-1.35) | 0.86(0.67-1.10) | 0.89(0.74-1.07) | 0.99(0.85-1.15) | 0.93(0.82-1.07) | 0.89(0.79-1.01) | Ref |  |
| adjusted OR (95% CI) ^**^ | 1.35(0.74-2.47) | 1.10(0.79-1.53) | 0.91(0.70-1.19) | 0.96(0.79-1.16) | 1.04(0.88-1.22) | 0.97(0.84-1.12) | 0.89(0.78-1.01) | Ref |  |
| **Total pregnancy loss** | 4/25(16.0) ^a^ | 25/88(28.4) ^a^ | 37/132(28.0) ^a^ | 63/255(24.7) ^a^ | 91/425(21.4) ^a^ | 122/598(20.4) ^a^ | 160/734(21.8) ^a^ | 419/1903(22.0) ^a^ | 0.386^*^ |
| OR (95% CI) | 0.68(0.23-1.98) | 1.41(0.87-2.26) | 1.38(0.93-2.05) | 1.16(0.86-1.58) | 0.97(0.75-1.25) | 0.91(0.72-1.14) | 0.99(0.80-1.21) | Ref |  |
| adjusted OR (95% CI) ^**^ | 0.62(0.20-1.93) | 1.26(0.78-2.04) | 1.39(0.92-2.11) | 1.10(0.81-1.51) | 0.90(0.69-1.18) | 0.86(0.69-1.09) | 0.95(0.77-1.17) | Ref |  |
| **Biochemical pregnancy loss** | 2/25(8.0) ^a^ | 12/88(13.6) ^a^ | 14/132(10.6) ^a^ | 25/255(9.8) ^a^ | 36/425(8.5) ^a^ | 46/598(7.7) ^a^ | 74/734(10.1) ^a^ | 180/1903(9.5) ^a^ | 0.650^*^ |
| OR (95% CI) | 0.83(0.20-3.56) | 1.51(0.81-2.83) | 1.14(0.64-2.02) | 1.04(0.67-1.62) | 0.89(0.61-1.29) | 0.80(0.57-1.12) | 1.07(0.81-1.43) | Ref |  |
| adjusted OR (95% CI) ^**^ | 0.71(0.16-3.20) | 1.35(0.72-2.53) | 1.12(0.62-2.03) | 1.01(0.65-1.57) | 0.87(0.59-1.29) | 0.77(0.54-1.08) | 1.07(0.80-1.43) | Ref |  |
| **Clinical pregnancy loss** | 2/23(8.7) ^a^ | 13/76(17.1) ^a^ | 23/118(19.5) ^a^ | 38/230(16.5) ^a^ | 55/389(14.1) ^a^ | 76/552(13.8) ^a^ | 86/660(13.0) ^a^ | 239/1723(13.9) ^a^ | 0.562^*^ |
| OR (95% CI) | 0.59(0.14-2.54) | 1.28(0.69-2.36) | 1.50(0.93-2.42) | 1.23(0.85-1.79) | 1.02(0.75-1.40) | 0.99(0.75-1.31) | 0.93(0.71-1.21) | Ref |  |
| adjusted OR (95% CI) ^**^ | 0.57(0.11-2.84) | 1.24(0.66-2.33) | 1.51(0.92-2.50) | 1.21(0.82-1.78) | 0.92(0.66-1.29) | 0.96(0.72-1.27) | 0.87(0.66-1.15) | Ref |  |
| **Live birth** | 21/45(46.7) ^a^ | 63/156(40.4) ^a^ | 95/260(36.0) ^a^ | 192/497(38.6) ^a^ | 334/796(42.0) ^a^ | 476/1161(41.0) ^a^ | 574/1426(40.3) ^a^ | 1484/3499(42.4) ^a^ | 0.419* |
| OR (95% CI) | 1.19(0.66-2.14) | 0.92(0.66-1.28) | 0.78(0.60-1.02) | 0.86(0.71-1.04) | 0.98(0.84-1.15) | 0.94(0.83-1.08） | 0.92(0.81-1.04) | Ref |  |
| adjusted OR (95% CI) ^**^ | 1.46(0.78-2.74) | 1.04(0.74-1.45) | 0.83(0.63-1.09) | 0.93(0.76-1.14) | 1.05(0.89-1.24) | 0.98(0.85-1.13) | 0.92(0.81-1.05) | Ref |  |

Qualitative data are presented as n (%).

OR, odds ratio; CI, confidence interval.

*χ2-test with Bonferroni adjustment; each superscript letter denotes a subset of Group categories whose column proportions do not differ significantly from each other at the 0.05 level.

** Analyses were adjusted for age, BMI, infertility duration, parity, main infertility cause, fertilization method, endometrial thickness, number of embryos transferred, embryo developmental stage, embryo quality at transfer of best embryo transferred, levels of E2, LH and P4 on the trigger day, E2 and P4 levels on the day of FET, number of oocytes retrieved and year of treatment.

**Supplementary Table S3 Pregnancy outcomes when treating dominant follicle size as a continuous variable.**

|  | **Regression coefficient** | **95% CI** | **P-value** |
| --- | --- | --- | --- |
| Positive pregnancy test | 0.004 | [-0.011-0.018] | 0.616 |
| Implantation | 0.002 | [-0.011-0.014] | 0.806 |
| Clinical pregnancy | 0.001 | [-0.013-0.016] | 0.886 |
| Total pregnancy loss | 0.001 | [-0.024-0.024] | 0.980 |
| Biochemical pregnancy loss | 0.013 | [-0.021-0.046] | 0.456 |
| Clinical pregnancy loss | -0.010 | [-0.041-0.020] | 0.505 |
| Live birth | 0.003 | [-0.012-0.018] | 0.673 |

Analyses were adjusted for age, BMI, infertility duration, parity, main infertility cause, fertilization method, FET cycle rank, endometrial thickness, number of embryos transferred, embryo developmental stage, embryo quality at transfer of best embryo transferred, levels of E2, LH and P4 on the trigger day, E2 and P4 levels on the day of FET, number of oocytes retrieved and year of treatment.

**Supplementary Table S4 Maternal complications of twin deliveries, according to dominant follicle size.**

| **Group** | **<12mm** | **12–12.9mm** | **13–13.9mm** | **14–14.9mm** | **15–15.9mm** | **16–16.9mm** | **17–17.9mm** | **≥18mm** | **P-value** |
| --- | --- | --- | --- | --- | --- | --- | --- | --- | --- |
| **Cycles (n)** | n=8 | n=19 | n=39 | n=76 | n=135 | n=187 | n=231 | n=577 |  |
| **Gestational diabetes mellitus** | 0(0) ^a^ | 3(15.8) ^a^ | 0(0) ^a^ | 8(10.5) ^a^ | 18(13.3) ^a^ | 18(9.6) ^a^ | 16(6.9) ^a^ | 52(9.0) ^a^ | 0.173^*^ |
| OR (95% CI) | NA | 1.55(0.81-2.98) | NA | 1.17(0.74-1.85) | 1.02(0.68-1.53) | 1.19(0.87-1.64) | 0.77(0.55-1.09) | Ref |  |
| adjusted OR (95% CI) ^**^ | NA | 1.46(0.74-2.86) | NA | 1.20(0.75-1.93) | 0.99(0.65-1.51) | 1.19(0.85-1.66) | 0.76(0.53-1.08) | Ref |  |
| **Hypertensive disorders of pregnancy** | 1(12.5) ^a^ | 0(0) ^a^ | 2(5.1) ^a^ | 6(7.9) ^a^ | 6(4.4) ^a^ | 9(4.8) ^a^ | 13(5.6) ^a^ | 34(5.9) ^a^ | 0.858^*^ |
| OR (95% CI) | 2.28(0.27-19.08) | NA | 0.86(0.20-3.73) | 1.37(0.56-3.38) | 0.74(0.31-1.81) | 0.81(0.38-1.72) | 0.95(0.49-1.84) | Ref |  |
| adjusted OR (95% CI) ^**^ | 1.20(0.16-8.83) | NA | 0.73(0.15-3.59) | 1.23(0.48-3.18) | 0.76(0.30-1.92) | 0.73(0.34-1.58) | 0.92(0.47-1.81) | Ref |  |
| **Abnormal-placentation** | 0(0) ^a^ | 0(0) ^a^ | 1(2.6) ^a^ | 3(3.9) ^a^ | 3(2.2) ^a^ | 3(1.6) ^a^ | 8(3.5) ^a^ | 10(1.7) ^a^ | 0.743^*^ |
| OR (95% CI) | NA | NA | 1.49(0.19-11.96) | 2.33(0.63-8.66) | 1.29(0.35-4.75) | 0.92(0.25-3.40) | 2.03(0.79-5.22) | Ref |  |
| adjusted OR (95% CI) ^**^ | NA | NA | 1.49(0.28-8.02) | 2.88(0.76-10.83) | 1.27(0.34-4.68) | 0.99(0.27-3.67) | 2.19(0.89-5.38) | Ref |  |
| **Preterm premature rupture of the membrane** | 1(12.5) ^a^ | 3(15.8) ^a^ | 4(10.3) ^a^ | 3(3.9) ^a^ | 15(11.1) ^a^ | 19(10.3) ^a^ | 33(14.3) ^a^ | 61(10.6) ^a^ | 0.411^*^ |
| OR (95% CI) | 1.21(0.15-9.99) | 1.59(0.45-5.60) | 0.97(0.33-2.81) | 0.35(0.11-1.14) | 1.06(0.58-1.92) | 0.96(0.56-1.65) | 1.41(0.90-2.22) | Ref |  |
| adjusted OR (95% CI) ^**^ | 1.14(0.17-7.76) | 1.92(0.55-6.77) | 1.06(0.35-3.24) | 0.36(0.11-1.23) | 1.30(0.68-2.47) | 0.93(0.50-1.74) | 1.55(0.95-2.52) | Ref |  |
| **Caesarean section** | 8(100.0) ^a^ | 19(100.0) ^a^ | 37(94.9) ^a^ | 74(97.4) ^a^ | 130(96.3) ^a^ | 182(97.3) ^a^ | 224(97.0) ^a^ | 555(96.2)^a^ | 0.949^*^ |
| OR (95% CI) | NA | NA | 0.73(0.17-3.24) | 1.47(0.34-6.36) | 1.03(0.38-2.77) | 1.44(0.54-3.87) | 1.27(0.53-3.01) | Ref |  |
| adjusted OR (95% CI) ^***^ | NA | NA | 1.09(0.32-3.73) | 1.64(0.37-7.26) | 1.46(0.55-3.87) | 1.69(0.61-4.67) | 1.72(0.64-4.61) | Ref |  |

Qualitative data are presented as n (%).

OR, odds ratio; CI, confidence interval.

Hypertensive disorders of pregnancy including pregnancy-induced hypertension and pre-eclampsia. Abnormal-placentation including placenta praevia and placental abruption.

*χ2-test with Bonferroni adjustment; each superscript letter denotes a subset of Group categories whose column proportions do not differ significantly from each other at the 0.05 level.

**Analyses were adjusted for age, BMI, infertility duration, parity, main infertility cause, fertilization method, FET cycle rank, endometrial thickness, number of embryos transferred, embryo developmental stage, embryo quality at transfer of best embryo transferred, levels of E2, LH and P4 on the trigger day, E2 and P4 levels on the day of FET, number of oocytes retrieved and year of treatment.

***Analyses were adjusted for Age, BMI, infertility duration, parity, main infertility cause, fertilization method, FET cycle rank, endometrial thickness, number of embryos transferred, embryo developmental stage, embryo quality at transfer of best embryo transferred, levels of E2, LH and P4 on the trigger day, E2 and P4 levels on the day of FET, number of oocytes retrieved and year of treatment, gestational diabetes, hypertensive disorder of pregnancy, abnormal placentation, preterm premature rupture of membranes.

**Supplementary Table S5 Perinatal outcomes of twin deliveries, according to dominant follicle size.**

| **Group** | **<12mm** | **12–12.9mm** | **13–13.9mm** | **14–14.9mm** | **15–15.9mm** | **16–16.9mm** | **17–17.9mm** | **≥18mm** | **P-value** |
| --- | --- | --- | --- | --- | --- | --- | --- | --- | --- |
| **Cycles (n)** | n=8 | n=19 | n=39 | n=76 | n=135 | n=187 | n=231 | n=577 |  |
| **Gestational age** | 35.38±3.46 | 36.32±1.16 | 35.95±2.25 | 36.25±1.51 | 35.96±1.77 | 36.14±1.92 | 36.08±1.84 | 36.11±1.83 | 0.859^*^ |
| **PTB** | 3(37.5) ^a^ | 9(47.4) ^a^ | 16(41.0) ^a^ | 34(44.7) ^a^ | 65(48.1) ^a^ | 81(43.3) ^a^ | 105(45.5) ^a^ | 270(46.8) ^a^ | 0.977^**^ |
| OR (95% CI) | 0.68(0.16-2.88) | 1.02(0.41-2.56) | 0.79(0.41-1.53) | 0.92(0.57-1.49) | 1.06(0.73-1.54) | 0.87(0.62-1.21) | 0.95(0.70-1.29) | Ref |  |
| adjusted OR (95% CI) ^***^ | 0.43(0.04-4.34) | 0.77(0.27-2.17) | 0.73(0.36-1.48) | 1.00(0.58-1.72) | 0.97(0.62-1.51) | 0.90(0.61-1.33) | 0.82(0.55-1.20) | Ref |  |
| **Babies born** | 16 | 38 | 78 | 152 | 270 | 374 | 462 | 1154 |  |
| **Sex** |  |  |  |  |  |  |  |  | 0.348^**^ |
| Female | 4(25.0) ^a^ | 17(44.7) ^a^ | 35(44.9) ^a^ | 69(45.4) ^a^ | 145(53.7) ^a^ | 184(49.2) ^a^ | 231(50.0) ^a^ | 570(49.4) ^a^ |  |
| Male | 12(75.0) ^a^ | 21(55.3) ^a^ | 43(55.1) ^a^ | 83(54.6) ^a^ | 125(46.3) ^a^ | 190(50.8) ^a^ | 231(50.0) ^a^ | 584(50.6) ^a^ |  |
| **Birthweight (g)** | 2413.13±560.32 | 2593.92±355.37 | 2501.14±508.10 | 2603.49±411.24 | 2501.48±434.59 | 2561.83±445.53 | 2538.24±418.88 | 2553.11±437.09 | 0.241^*^ |
| **LBW (<2500g)** | 6(37.5) ^a^ | 12(31.6) ^a^ | 36(46.2) ^a^ | 52(34.2) ^a^ | 125(46.3) ^a^ | 133(35.6) ^a^ | 194(42.0) ^a^ | 444(38.5) ^a^ | 0.059^**^ |
| OR (95% CI) | 0.96(0.35-2.66) | 0.74(0.37-1.48) | 1.37(0.87-2.17) | 0.83(0.58-1.19) | 1.38(1.06-1.80) | 0.88(0.69-1.13) | 1.16(0.93-1.44) | Ref |  |
| adjusted OR (95% CI) ^****^ | 1.01(0.29-3.53) | 0.65(0.31-1.37) | 1.51(0.79-2.91) | 0.91(0.59-1.42) | 1.39(0.97-1.99) | 0.92(0.66-1.28) | 1.13(0.85-1.51) | Ref |  |
| **SGA** | 0(0) ^a^ | 2(5.3) ^a^ | 6(7.7) ^a^ | 7(4.6) ^a^ | 13(4.8) ^a^ | 20(5.3) ^a^ | 20(4.3) ^a^ | 66(5.7) ^a^ | 0.851^**^ |
| OR (95% CI) | NA | 0.92(0.22-3.89) | 1.37(0.58-3.28) | 0.80(0.36-1.77) | 0.83(0.45-1.54) | 0.93(0.56-1.56) | 0.75(0.45-1.25) | Ref |  |
| adjusted OR (95% CI) ^***^ | NA | 1.05(0.13-8.37) | 1.39(0.54-3.61) | 0.79(0.34-1.84) | 0.90(0.46-1.78) | 0.95(0.53-1.70) | 0.74(0.44-1.23) | Ref |  |
| **LGA** | 1(6.3) ^a^ | 7(18.4) ^a^ | 10(12.8) ^a^ | 33(21.7) ^a^ | 43(15.9) ^a^ | 65(17.4) ^a^ | 87(18.8) ^a^ | 213(18.5) ^a^ | 0.589^**^ |
| OR (95% CI) | 0.30(0.04-2.24) | 0.99(0.43-2.30) | 0.65(0.33-1.28) | 1.23(0.81-1.85) | 0.84(0.59-1.20) | 0.93(0.68-1.26) | 1.03(0.78-1.35) | Ref |  |
| adjusted OR (95% CI) ^***^ | 0.28(0.04-1.84) | 1.08(0.35-3.31) | 0.64(0.29-1.45) | 1.20(0.76-1.91) | 0.87(0.56-1.36) | 0.92(0.65-1.30) | 1.09(0.78-1.52) | Ref |  |

* One-way ANOVA.

OR, odds ratio; CI, confidence interval; PTB, preterm birth; LBW, low birthweight; SGA, small for gestational age; LGA, large for gestational age.

**χ2-test with Bonferroni adjustment; each superscript letter denotes a subset of Group categories whose column proportions do not differ significantly from each other at the 0.05 level.

***Analyses were adjusted for age, BMI, infertility duration, parity, main infertility cause, fertilization method, FET cycle rank, endometrial thickness, number of embryos transferred, embryo developmental stage, embryo quality at transfer of best embryo transferred, levels of E2, LH and P4 on the trigger day, E2 and P4 levels on the day of FET, number of oocytes retrieved and year of treatment, gestational diabetes, hypertensive disorder of pregnancy, abnormal placentation, preterm premature rupture of membranes.

****Analyses were adjusted for age, BMI, infertility duration, parity, main infertility cause, fertilization method, FET cycle rank, endometrial thickness, number of embryos transferred, embryo developmental stage, embryo quality at transfer of best embryo transferred, levels of E2, LH and P4 on the trigger day, E2 and P4 levels on the day of FET, number of oocytes retrieved and year of treatment, gestational diabetes, hypertensive disorder of pregnancy, abnormal placentation, preterm premature rupture of membranes and sex of the infant.

**Supplementary Table S6 Main reproductive outcomes of nulliparous women, according to dominant follicle size.**

| **Group** | **<12mm** | **12–12.9mm** | **13–13.9mm** | **14–14.9mm** | **15–15.9mm** | **16–16.9mm** | **17–17.9mm** | **≥18mm** | **P value** |
| --- | --- | --- | --- | --- | --- | --- | --- | --- | --- |
| **Cycles (n)** | 85 | 267 | 431 | 772 | 1187 | 1807 | 2183 | 5835 |  |
| **Positive pregnancy test** | 45/85(52.9) ^a^ | 134/267(50.2) ^a^ | 208/431(48.3) ^a^ | 380/772(49.2) ^a^ | 593/1187(50.0) ^a^ | 898/1807(49.7) ^a^ | 1083/2183(49.6) ^a^ | 3024/5835(51.8) ^a^ | 0.443^*^ |
| OR (95% CI) | 1.05(0.68-1.61) | 0.94(0.73-1.20) | 0.87(0.71-1.06) | 0.90(0.78-1.05) | 0.93(0.82-1.05) | 0.92(0.83-1.02) | 0.92(0.83-1.01） | Ref |  |
| adjusted OR (95% CI) ^**^ | 1.24(0.80-1.93) | 1.03(0.80-1.32) | 0.92(0.75-1.13) | 0.98(0.83-1.15) | 0.95(0.83-1.08) | 0.94(0.84-1.05) | 0.90(0.81-1.00) | Ref |  |
| **Implantation** | 53/147(36.1) ^a^ | 144/474(30.4) ^a^ | 234/759(30.8) ^a^ | 423/1360(31.1) ^a^ | 678/2094(32.4) ^a^ | 1019/3171(32.1) ^a^ | 1227/3892(31.5) ^a^ | 3391/10252(33.1) ^a^ | 0.401^*^ |
| OR (95% CI) | 1.14(0.81-1.60) | 0.88(0.72-1.08) | 0.90(0.77-1.06) | 0.92(0.81-1.03) | 0.97(0.88-1.07) | 0.96(0.89-1.04) | 0.93(0.86-1.01) | Ref |  |
| adjusted OR (95% CI) ^**^ | 1.31(0.89-1.92) | 0.97(0.79-1.20) | 0.96(0.80-1.15) | 0.98(0.86-1.12) | 0.98(0.88-1.10) | 0.97(0.88-1.07) | 0.93(0.85-1.02) | Ref |  |
| **Clinical pregnancy** | 41/85(48.2) ^a^ | 121/267(45.3) ^a^ | 188/431(43.6) ^a^ | 350/772(45.3) ^a^ | 532/1187(44.8) ^a^ | 815/1807(45.1) ^a^ | 970/2183(44.4) ^a^ | 2724/5835(46.7) ^a^ | 0.618^*^ |
| OR (95% CI) | 1.06(0.69-1.63) | 0.95(0.74-1.21) | 0.88(0.73-1.08) | 0.95(0.82-1.10) | 0.93(0.82-1.05) | 0.94(0.84-1.04) | 0.91(0.83-1.01) | Ref |  |
| adjusted OR (95% CI) ^**^ | 1.25(0.80-1.96) | 1.03(0.80-1.32) | 0.94(0.76-1.16) | 1.03(0.88-1.21) | 0.95(0.83-1.08) | 0.96(0.86-1.08) | 0.90(0.81-1.00) | Ref |  |
| **Total pregnancy loss** | 10/45(22.2) ^a^ | 31/134(23.1) ^a^ | 47/208(22.6) ^a^ | 89/380(23.4) ^a^ | 139/593(23.4) ^a^ | 191/898(21.3) ^a^ | 251/1083(23.2) ^a^ | 659/3024(21.8) ^a^ | 0.944^*^ |
| OR (95% CI) | 1.03(0.51-2.08) | 1.08(0.72-1.63) | 1.05(0.75-1.47) | 1.10(0.85-1.41) | 1.10(0.89-1.35) | 0.97(0.81-1.16) | 1.08(0.92-1.28) | Ref |  |
| adjusted OR (95% CI) ^**^ | 1.00(0.49-2.03) | 1.07(0.70-1.62) | 1.03(0.73-1.46) | 1.04(0.80-1.36) | 0.94(0.78-1.13) | 0.94(0.78-1.13) | 1.06(0.89-1.25) | Ref |  |
| **Biochemical pregnancy loss** | 4/45(8.9) ^a^ | 13/134(9.7) ^a^ | 20/208(9.6) ^a^ | 30/380(7.9) ^a^ | 61/593(10.3) ^a^ | 83/898(9.2) ^a^ | 113/1083(10.4) ^a^ | 300/3024(9.9) ^a^ | 0.917^*^ |
| OR (95% CI) | 0.89(0.32-2.49) | 0.98(0.54-1.75) | 0.97(0.60-1.56) | 0.78(0.53-1.15) | 1.04(0.78-1.39) | 0.93(0.72-1.19) | 1.06(0.84-1.33) | Ref |  |
| adjusted OR (95% CI) ^**^ | 0.87(0.31-2.47) | 0.99(0.55-1.78) | 0.95(0.59-1.55) | 0.75(0.50-1.12) | 1.02(0.76-1.38) | 0.89(0.69-1.16) | 1.04(0.83-1.31) | Ref |  |
| **Clinical pregnancy loss** | 6/41(14.6) ^a^ | 18/121(14.9) ^a^ | 27/188(14.4) ^a^ | 59/350(16.9) ^a^ | 78/532(14.7) ^a^ | 108/815(13.3) ^a^ | 138/970(14.2) ^a^ | 359/2724(13.2) ^a^ | 0.720^*^ |
| OR (95% CI) | 1.13(0.47-2.70) | 1.15(0.69-1.92) | 1.11(0.72-1.69) | 1.34(0.99-1.81) | 1.13(0.87-1.48) | 1.01(0.80-1.27) | 1.09(0.88-1.35) | Ref |  |
| adjusted OR (95% CI) ^**^ | 1.14(0.47-2.77) | 1.11(0.65-1.88) | 1.08(0.70-1.68) | 1.29(0.94-1.77) | 1.10(0.83-1.45) | 0.99(0.78-1.25) | 1.07(0.86-1.33) | Ref |  |
| **Live birth** | 35/85(41.2) ^a^ | 103/267(38.6) ^a^ | 161/431(37.4) ^a^ | 291/772(37.7) ^a^ | 454/1187(38.2) ^a^ | 707/1807(39.1) ^a^ | 832/2183(38.1) ^a^ | 2365/5835(40.5) ^a^ | 0.404* |
| OR (95% CI) | 1.03(0.67-1.59) | 0.92(0.72-1.19) | 0.88(0.72-1.07) | 0.89(0.76-1.04) | 0.91(0.80-1.03) | 0.94(0.85-1.05） | 0.90(0.82-1.00) | Ref |  |
| adjusted OR (95% CI) ^**^ | 1.19(0.76-1.86) | 1.01(0.78-1.31) | 0.93(0.75-1.15) | 0.97(0.82-1.14) | 0.93(0.82-1.07) | 0.97(0.87-1.09) | 0.89(0.80-1.00) | Ref |  |

Qualitative data are presented as n (%).

OR, odds ratio; CI, confidence interval.

*χ2-test with Bonferroni adjustment; each superscript letter denotes a subset of Group categories whose column proportions do not differ significantly from each other at the 0.05 level.

** Analyses were adjusted for age, BMI, infertility duration, main infertility cause, fertilization method, FET cycle rank, endometrial thickness, number of embryos transferred, embryo developmental stage, embryo quality at transfer of best embryo transferred, levels of E2, LH and P4 on the trigger day, E2 and P4 levels on the day of FET, number of oocytes retrieved and year of treatment.

**Supplementary Table S7 Maternal complications of the singleton newborns born to nulliparous women, according to dominant follicle size.**

| **Group** | **<12mm** | **12–12.9mm** | **13–13.9mm** | **14–14.9mm** | **15–15.9mm** | **16–16.9mm** | **17–17.9mm** | **≥18mm** | **P-value** |
| --- | --- | --- | --- | --- | --- | --- | --- | --- | --- |
|  | n=27 | n=84 | n=123 | n=225 | n=333 | n=539 | n=629 | n=1837 |  |
| **Gestational diabetes mellitus** | 0(0) ^a^ | 11(13.1) ^a^ | 7(5.7) ^a^ | 23(10.2) ^a^ | 30(9.0) ^a^ | 56(10.4) ^a^ | 44(7.0) ^a^ | 163(8.9) ^a^ | 0.144^*^ |
| OR (95% CI) | NA | 1.55(0.81-2.98) | 0.62(0.28-1.35) | 1.17(0.74-1.85) | 1.02(0.68-1.53) | 1.19(0.87-1.64) | 0.77(0.55-1.09) | Ref |  |
| adjusted OR (95% CI) ^**^ | NA | 1.46(0.74-2.86) | 0.60(0.27-1.34) | 1.20(0.75-1.93) | 0.99(0.65-1.51) | 1.19(0.85-1.66) | 0.76(0.53-1.08) | Ref |  |
| **Hypertensive disorders of pregnancy** | 0(0) ^a^ | 3(3.6) ^a^ | 2(1.6) ^a^ | 8(3.6) ^a^ | 5(1.5) ^a^ | 6(1.1) ^a^ | 14(2.2) ^a^ | 33(1.8) ^a^ | 0.352^*^ |
| OR (95% CI) | NA | 2.03(0.61-6.74) | 0.90(0.21-3.81) | 2.02(0.92-4.42) | 0.83(0.32-2.15) | 0.62(0.26-1.48) | 1.24(0.66-2.34) | Ref |  |
| adjusted OR (95% CI) ^**^ | NA | 1.28(0.34-4.79) | 0.61(0.12-2.99) | 1.97(0.90-4.31) | 0.81(0.30-2.15) | 0.67(0.27-1.68) | 1.24(0.63-2.40) | Ref |  |
| **Abnormal-placentation** | 1(3.7) ^a^ | 4(4.8) ^a^ | 5(4.1) ^a^ | 6(2.7) ^a^ | 9(2.7) ^a^ | 16(3.0) ^a^ | 15(2.4) ^a^ | 34(1.9) ^a^ | 0.426^*^ |
| OR (95% CI) | 2.04(0.27-15.47) | 2.65(0.92-7.65) | 2.25(0.86-5.85) | 1.45(0.60-3.50) | 1.47(0.70-3.10) | 1.62(0.89-2.96) | 1.30(0.70-2.40) | Ref |  |
| adjusted OR (95% CI) ^**^ | 1.65(0.19-14.78) | 2.04(0.69-6.02) | 1.63(0.62-4.27) | 1.08(0.44-2.62) | 1.26(0.59-2.70) | 1.37(0.73-2.59) | 1.18(0.62-2.24) | Ref |  |
| **Preterm premature rupture of the membrane** | 0(0) ^a^ | 3(3.6) ^a^ | 2(1.6) ^a^ | 7(3.1) ^a^ | 7(2.1) ^a^ | 7(1.3) ^a^ | 8(1.3) ^a^ | 28(1.5) ^a^ | 0.434^*^ |
| OR (95% CI) | NA | 2.39(0.71-8.04) | 1.07(0.25-4.54) | 2.08(0.90-4.81) | 1.39(0.60-3.20) | 0.85(0.37-1.96) | 0.83(0.38-1.84) | Ref |  |
| adjusted OR (95% CI) ^**^ | NA | 2.31(0.62-8.57) | 0.99(0.21-4.67) | 2.10(0.85-5.15) | 1.62(0.70-3.77) | 0.98(0.41-2.34) | 0.87(0.38-2.02) | Ref |  |
| **Caesarean section** | 21(77.8) ^a^ | 57(67.9) ^a^ | 93(75.6) ^a^ | 155(68.9) ^a^ | 246(73.9) ^a^ | 349(64.7) ^a^ | 418(66.5) ^a^ | 1256(68.4) ^a^ | 0.069^*^ |
| OR (95% CI) | 1.62(0.65-4.03) | 0.98(0.61-1.56) | 1.43(0.94-2.19) | 1.02(0.76-1.38) | 1.31(1.00-1.70) | 0.85(0.69-1.04) | 0.92(0.76-1.11) | Ref |  |
| adjusted OR (95% CI) ^***^ | 1.73(0.69-4.29) | 0.88(0.52-1.46) | 1.40(0.90-2.18) | 0.99(0.73-1.45) | 1.30(0.98-1.72) | 0.84(0.68-1.04) | 0.88(0.72-1.07) | Ref |  |

Qualitative data are presented as n (%).

OR, odds ratio; CI, confidence interval.

Hypertensive disorders of pregnancy including pregnancy-induced hypertension and pre-eclampsia. Abnormal-placentation including placenta praevia and placental abruption.

*χ2-test with Bonferroni adjustment; each superscript letter denotes a subset of Group categories whose column proportions do not differ significantly from each other at the 0.05 level.

**Analyses were adjusted for age, BMI, infertility duration, parity, main infertility cause, fertilization method, FET cycle rank, endometrial thickness, number of embryos transferred, embryo developmental stage, embryo quality at transfer of best embryo transferred, levels of E2, LH and P4 on the trigger day, E2 and P4 levels on the day of FET, number of oocytes retrieved and year of treatment.

***Analyses were adjusted for Age, BMI, infertility duration,, main infertility cause, fertilization method, FET cycle rank, endometrial thickness, number of embryos transferred, embryo developmental stage, embryo quality at transfer of best embryo transferred, levels of E2, LH and P4 on the trigger day, E2 and P4 levels on the day of FET, number of oocytes retrieved and year of treatment, gestational diabetes, hypertensive disorder of pregnancy, abnormal placentation, preterm premature rupture of membranes.

**Supplementary Table S8 Perinatal outcomes of singleton newborns born to nulliparous women, according to dominant follicle size.**

| **Group** | **<12mm** | **12–12.9mm** | **13–13.9mm** | **14–14.9mm** | **15–15.9mm** | **16–16.9mm** | **17–17.9mm** | **≥18mm** | **P-value** |
| --- | --- | --- | --- | --- | --- | --- | --- | --- | --- |
|  | n=27 | n=84 | n=123 | n=225 | n=333 | n=539 | n=629 | n=1837 |  |
| **Gestational age** | 38.33±2.48 | 38.51±1.65 | 38.66±1.57 | 38.45±1.64 | 38.68±1.43 | 38.70±1.36 | 38.65±1.46 | 38.59±1.48 | 0.383^*^ |
| **Birthweight (g)** | 3360.19±635.78 | 3327.98±521.34 | 3396.12±480.61 | 3281.77±513.74 | 3387.04±472.50 | 3316.51±463.73 | 3331.34±449.90 | 3357.04±476.58 | 0.108^*^ |
| **Sex** |  |  |  |  |  |  |  |  | 0.885^**^ |
| Female | 12(44.4) ^a^ | 38(45.2) ^a^ | 59(48.0) ^a^ | 116(51.6) ^a^ | 171(51.4) ^a^ | 266(49.4) ^a^ | 299(47.5) ^a^ | 883(48.1) ^a^ |  |
| Male | 15(55.6) ^a^ | 46(54.8) ^a^ | 64(52.0) ^a^ | 109(48.4) ^a^ | 162(48.6) ^a^ | 273(50.6) ^a^ | 330(52.5) ^a^ | 954(51.9) ^a^ |  |
| **PTB** | 2(7.4) ^a^ | 5(6.0) ^a^ | 6(4.9) ^a^ | 17(7.6) ^a^ | 19(5.7) ^a^ | 22(4.1) ^a^ | 27(4.3) ^a^ | 97(5.3) ^a^ | 0.591^**^ |
| OR (95% CI) | 1.44(0.34-6.15) | 1.14(0.45-2.87) | 0.92(0.40-2.14) | 1.47(0.86-2.50) | 1.09(0.65-1.80) | 0.76(0.48-1.23) | 0.81(0.52-1.25) | Ref |  |
| adjusted OR (95% CI) ^***^ | 2.42(0.61-9.56) | 0.45(0.14-1.42) | 0.65(0.25-1.69) | 0.90(0.45-1.81) | 0.84(0.43-1.63) | 0.66(0.36-1.18) | 0.63(0.37-1.08) | Ref |  |
| **LBW (<2500g)** | 2(7.4) ^a^ | 2(2.4) ^a^ | 6(4.9) ^a^ | 13(5.8) ^a^ | 11(3.3) ^a^ | 18(3.3) ^a^ | 21(3.3) ^a^ | 64(3.5) ^a^ | 0.598^**^ |
| OR (95% CI) | 2.22(0.51-9.56) | 0.68(0.16-2.81) | 1.42(0.60-3.35) | 1.70(0.92-3.14) | 0.95(0.50-1.81) | 0.96(0.56-1.63) | 0.96(0.58-1.58) | Ref |  |
| adjusted OR (95% CI) ^***^ | 3.41(0.86-13.50) | 0.53(0.10-2.71) | 1.26(0.54-2.92) | 1.25(0.62-2.54) | 0.84(0.40-1.76) | 0.96(0.53-1.72) | 0.86(0.51-1.46) | Ref |  |
| **Macrosomia (≥4000g)** | 4(14.8) ^a^ | 7(8.3) ^a^ | 10(8.1) ^a^ | 15(6.7) ^a^ | 29(8.7) ^a^ | 39(7.2) ^a^ | 47(7.5) ^a^ | 149(8.1) ^a^ | 0.869^**^ |
| OR (95% CI) | 1.97(0.67-5.77) | 1.03(0.47-2.27) | 1.00(0.51-1.96) | 0.81(0.47-1.40) | 1.08(0.71-1.64) | 0.88(0.61-1.28) | 0.92(0.65-1.29) | Ref |  |
| adjusted OR (95% CI) ^***^ | 2.01(0.66-6.15) | 0.83(0.37-1.89) | 0.90(0.44-1.82) | 0.81(0.46-1.42) | 1.04(0.69-1.58) | 0.90(0.62-1.30) | 0.90(0.63-1.28) | Ref |  |
| **SGA** | 0(0) ^a^ | 6(7.1) ^a^ | 4(3.3) ^a^ | 11(4.9) ^a^ | 14(4.2) ^a^ | 31(5.8) ^a^ | 24(3.8) ^a^ | 85(4.6) ^a^ | 0.574^**^ |
| OR (95% CI) | NA | 1.59(0.67-3.74) | 0.70(0.25-1.92) | 1.06(0.56-2.02) | 0.91(0.51-1.61) | 1.26(0.82-1.92) | 0.82(0.52-1.30) | Ref |  |
| adjusted OR (95% CI) ^***^ | NA | 2.01(0.86-4.68) | 0.76(0.24-2.38) | 1.09(0.55-2.14) | 1.03(0.56-1.90) | 1.35(0.87-2.10) | 0.81(0.50-1.32) | Ref |  |
| **LGA** | 6(22.2) ^a^ | 13(15.5) ^a^ | 19(15.4) ^a^ | 26(11.6) ^a^ | 53(15.9) ^a^ | 71(13.2) ^a^ | 85(13.5) ^a^ | 298(16.2) ^a^ | 0.325^**^ |
| OR (95% CI) | 1.48(0.59-3.69) | 0.95(0.52-1.73) | 0.94(0.57-1.56) | 0.68(0.44-1.03) | 0.98(0.71-1.35) | 0.78(0.59-1.04) | 0.81(0.62-1.05) | Ref |  |
| adjusted OR (95% CI) ^***^ | 1.59(0.61-4.16) | 0.77(0.41-1.46) | 0.85(0.51-1.42) | 0.66(0.43-1.03) | 0.92(0.66-1.29) | 0.78(0.58-1.04) | 0.77(0.59-1.01) | Ref |  |

* One-way ANOVA.

OR, odds ratio; CI, confidence interval; PTB, preterm birth; LBW, low birthweight; SGA, small for gestational age; LGA, large for gestational age.

**χ2-test with Bonferroni adjustment; each superscript letter denotes a subset of Group categories whose column proportions do not differ significantly from each other at the 0.05 level.

***Analyses were adjusted for age, BMI, infertility duration, main infertility cause, fertilization method, FET cycle rank, endometrial thickness, number of embryos transferred, embryo developmental stage, embryo quality at transfer of best embryo transferred, levels of E2, LH and P4 on the trigger day, E2 and P4 levels on the day of FET, number of oocytes retrieved and year of treatment, gestational diabetes, hypertensive disorder of pregnancy, abnormal placentation, preterm premature rupture of membranes.

**Supplementary Table S9 The distribution of cases according to dominant follicle size by year (excluding data aggregation for follicle sized ≥18mm).**

|  | **10–10.9mm** | **11–11.9mm** | **12–12.9mm** | **13–13.9mm** | **14–14.9mm** | **15–15.9mm** | **16–16.9mm** | **17–17.9mm** | **Total** |
| --- | --- | --- | --- | --- | --- | --- | --- | --- | --- |
| 2013 | 0 | 8 | 27 | 58 | 146 | 207 | 309 | 343 | 1098 |
| 2014 | 3 | 6 | 59 | 76 | 154 | 256 | 381 | 440 | 1375 |
| 2015 | 7 | 9 | 80 | 108 | 163 | 245 | 345 | 468 | 1425 |
| 2016 | 11 | 14 | 57 | 94 | 138 | 214 | 308 | 406 | 1242 |
| 2017 | 5 | 9 | 41 | 59 | 97 | 142 | 230 | 265 | 848 |
| 2018 | 0 | 8 | 16 | 28 | 52 | 90 | 122 | 175 | 491 |
| 2019 | 5 | 8 | 16 | 32 | 56 | 84 | 134 | 171 | 506 |
| 2020 | 1 | 2 | 4 | 14 | 48 | 51 | 72 | 88 | 280 |
| 2021 | 0 | 3 | 1 | 7 | 23 | 33 | 64 | 65 | 196 |
| 2022 | 1 | 1 | 2 | 5 | 16 | 23 | 45 | 46 | 139 |
| 2023 | 1 | 0 | 2 | 5 | 15 | 33 | 43 | 48 | 147 |
| **Total** | 34 | 68 | 305 | 486 | 908 | 1378 | 2053 | 2515 | 7747 |
